# Patterns of Coral-Reef Finfish Species Disappearances Inferred from Fishers’ Knowledge in Global Epicentre of Marine Shorefish Diversity

**DOI:** 10.1371/journal.pone.0155752

**Published:** 2016-05-18

**Authors:** Margarita N. Lavides, Erina Pauline V. Molina, Gregorio E. de la Rosa, Aileen C. Mill, Stephen P. Rushton, Selina M. Stead, Nicholas V. C. Polunin

**Affiliations:** 1 Haribon Foundation for the Conservation of Natural Resources, Aurora Blvd Quezon City, 1102, Philippines; 2 School of Marine Science & Technology, Newcastle University, Newcastle upon Tyne, NE17RU, United Kingdom; 3 School of Biology, Newcastle University, Newcastle upon Tyne, NE17RU, United Kingdom; Department of Agriculture and Water Resources, AUSTRALIA

## Abstract

In the Philippines, very high fishing pressure coincides with the globally greatest number of shorefish species, yet no long-term fisheries data are available to explore species-level changes that may have occurred widely in the most species rich and vulnerable marine ecosystem, namely coral reefs. Through 2655 face-to-face interviews conducted between August 2012 and July 2014, we used fishers’ recall of past catch rates of reef-associated finfish to infer species disappearances from catches in five marine key biodiversity areas (Lanuza Bay, Danajon Bank, Verde Island Passage, Polillo Islands and Honda Bay). We modeled temporal trends in perceived catch per unit effort (CPUE) based on fishers’ reports of typical good days’ catches using Generalized Linear Mixed Modelling. Fifty-nine different finfish disappeared from catches between the 1950s and 2014; 42 fish were identified to species level, two to genus, seven to family and eight to local name only. Five species occurring at all sites with the greatest number of fishers reporting zero catches were the green bumphead parrotfish (*Bolbometopon muricatum*), humphead wrasse (*Cheilinus undulatus*), African pompano (*Alectis ciliaris*), giant grouper (*Epinephelus lanceolatus*) and mangrove red snapper (*Lutjanus argentimaculatus*). Between the 1950s and 2014, the mean perceived CPUE of bumphead parrotfish declined by 88%, that of humphead wrasse by 82%, African pompano by 66%, giant grouper by 74% and mangrove red snapper by 64%. These declines were mainly associated with excess and uncontrolled fishing, fish life-history traits like maximum body size and socio-economic factors like access to market infrastructure and services, and overpopulation. The fishers’ knowledge is indicative of extirpations where evidence for these losses was otherwise lacking. Our models provide information as basis for area-based conservation and regional resource management particularly for the more vulnerable, once common, large, yet wide-ranging reef finfish species.

## Introduction

Coral reefs occupy less than one percent of the marine area, but they are home to 25 percent of all known marine fish species [1–3]. In the Philippines, part of the world’s most biodiverse marine environment [4, 5] and anthropogenically-threatened marine region [6], coral reefs support around 20% of total marine fisheries production [3, 7]. Fishery products provide 11.7% of total Filipino food consumption [8] and in 2010 5–6 million Filipinos depended directly on the fishing industry for livelihoods [9, 10]. More than one million ‘municipal’ fishers (vessels ≤3 gross tonnes, GT) exploit coral reefs and adjacent shallow marine ecosystems. This has led to habitat degradation and a halving of the potential Philippine coral-reef fishery production by the 1990s [7, 11]. Philippine fishing grounds that were very productive in the 1950s and 1960s [12] were overfished by the 1980s [13] and two-thirds of the major fishing grounds are now overfished [9, 14]. In addition to high fishing pressure, other factors such as life-history traits of the species targeted and the socio-economic circumstances of the fishers have contributed to this poor state of the fisheries [15].

Demonstrating declines in Philippine reef fisheries is especially difficult because landings data are aggregated across the marine ecosystems in overall ‘municipal’ fisheries production [7] and the numerous landing sites make data gathering logistically challenging, costly and time consuming. In addition, Philippine reef fishery science is of recent origin [16–19]. Scientific trawl data from habitats other than coral reefs indicate substantial declines in total biomass of demersal fishes [20] and national underwater visual surveys of reef fishes revealed that 97% of large-bodied species have low abundances and/or restricted distribution [21]; data from Central Visayas suggest that some species may have disappeared [22]. However, there has been no attempt to investigate any large-scale declines or losses of coral reef species.

We used anecdotal information gathered systematically from fishers (hereafter fishers’ knowledge) [23] to infer marine finfish disappearances [24–38] because this is the only available information source on catch trends of the ca. 1658 Philippine reef fish species over the past 40–60 years. Fishers’ recollections have indicated declines in catch rates in small-scale Philippine fisheries [39], but these are to species level only for reef-associated fishes at two small islands [27] and seahorses at one island [40] off Bohol. Our study is the first to use fishers’ knowledge and robust ecological modelling techniques to identify reef fish species that may be vulnerable to extirpation in five Philippine marine Key Biodiversity Areas (KBAs). Marine KBAs are priority sites for conservation where (1) one or more globally threatened species are present and (2) there are species where at least 1% of the estimated global population gathers during some life stage [41, 42].

Here we explore factors such as fishery characteristics and practices (e.g. boat engine power, years’ fishing experience, gear selection)[27, 43, 44], fish life-history traits [27, 44–49], and socio-economic factors [50, 51] that might be linked with reef finfish species extirpations. Linear mixed modelling allowed us to test relationships of perceived CPUE with relevant variables. The paper further examines proxies of life-history such as maximum body size which are linked with fish vulnerability to depletion [27, 44–49]. Selected socio-economic factors such as human population size, market access and availability of community infrastructure and services were tested in addition as predictors of finfish depletion [50, 51].

## Methods

### Ethics statement

This study was approved by Newcastle University and the Haribon Foundation’s Board of Trustees ethical procedures, both of which considered work with human subjects. Permission to gather the data was verbally granted by the existing Peoples’ Organization and current village (barangay) captain in each village visited and all study participants consented to inclusion in the study. For Honda Bay (Palawan), a required application to conduct the study in the area was granted by the Palawan Council for Sustainable Development.

### Study areas

**Fig 1:**
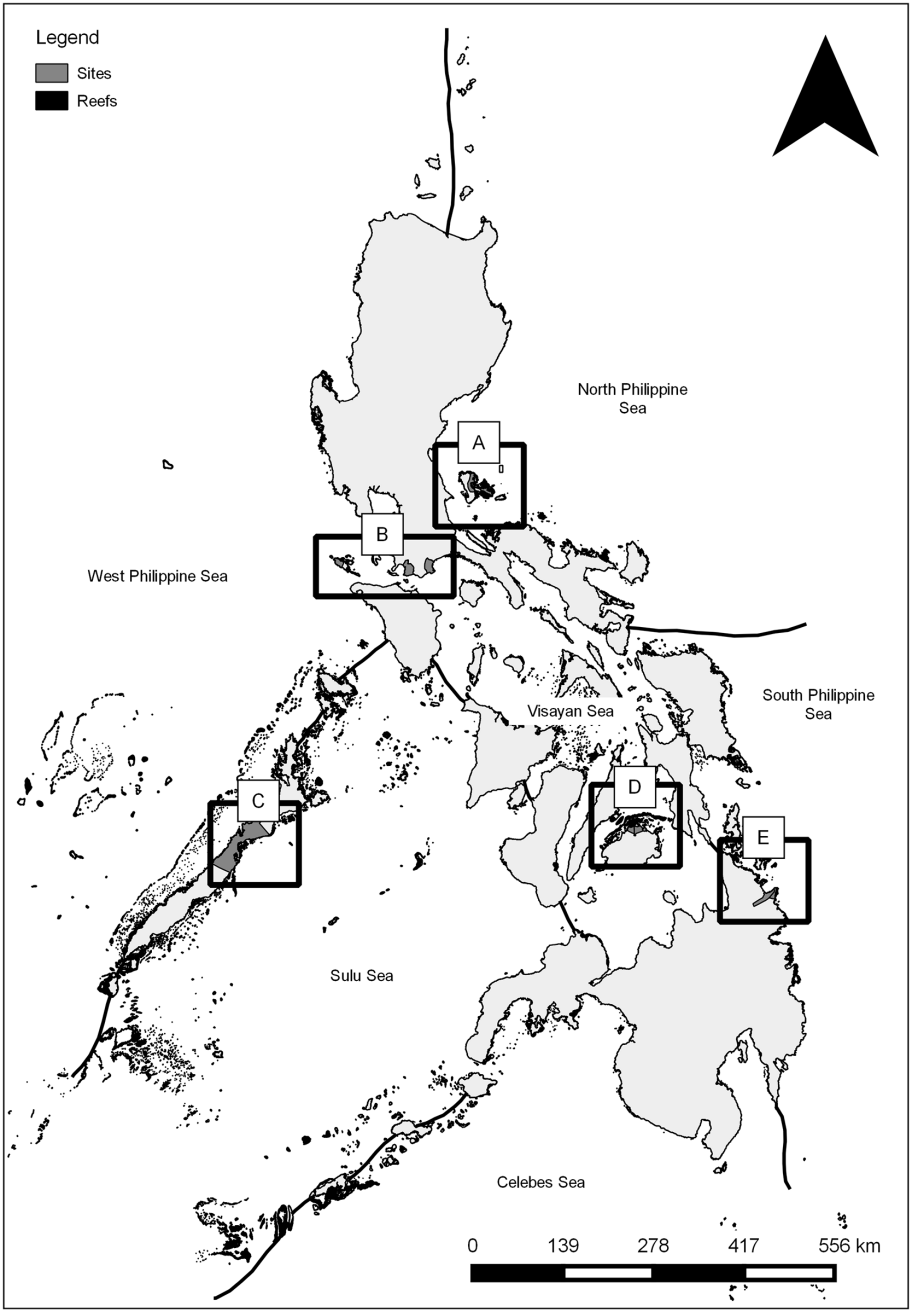
Map showing location of study areas. A = Polillo Islands; B = Verde Island Passage; C = Honda Bay; D = Danajon Bank; E = Lanuza Bay.

Study areas representing five of six Philippine marine biogeographic zones were selected based on their status as marine Key Biodiversity Areas (KBAs)[41, 42] containing a major fishing ground in the country or region and having sources of historical or current fish visual census or landings data (Fig 1). Fishers were interviewed in a total of 61 villages in the five marine KBAs: 14 villages in Lanuza Bay and 18 villages on Danajon Bank in August-November 2012; 14 villages in Verde Island Passage and 10 villages in Polillo Islands in March-July 2013; and 5 villages in Honda Bay in July 2014.

### Fisher interviews

Snowball sampling, that relies on referrals from initial subjects to generate additional interviewees [52], was used to maximize the number of interviews accomplished per day since people knew who else was present in the village each day. Fishers who were 21 years old and above were invited to be interviewed face-to-face. Interviewees were placed in three groups based on their age (early [21–41 years]), mid [42–62 years] and late [≥63 years]) (S1 Table). We targeted at least 15% of the total fisher population.

A semi-structured interview was used to acquire data on fishers’ recall of their typical good days’ catches at present and in each of the decades 1950s, 1960s, 1970s, 1980s, 1990s, and 2000s [27]. Fishers were also asked to identify finfish species that had once been, but were no longer captured and asked what their recollections were of these species’ catch per unit effort (CPUE) in each decade based on a typical good day’s catch [27] (S2 Table).

Significant Philippine events were used to help memory of the periods involved [27,40]. These were the period after the Japanese occupation (1950s), and the political landmarks of the presidents who were governing during the decade (Magsaysay [1960s], Marcos [1970s], Cory [1980s], Ramos [1990s], and Erap and Gloria [2000s]). Different local names used for the same fish species were reconciled by cross-referencing with photographic field guides, including those specific to the site [53–56] and with FishBase [57]. Because fishers can have multiple names for the same species, or single names for several species, caution was exercised in the use and validation of local names, particularly at species level.

Potential issues in interpreting abundance trends based on fishers’ recollections of past catches include the limitations of CPUE as a measure of abundance [52, 58] and psychological biases [40, 59]. Fishers may remember good days’ catches better than normal days’ catches [59] and older people with expertise derived from a lifetime of personal experiences [60–64], such as in fishing, may have good retention of fishing experiences even when memory of other matters is severely curtailed [60–64]. Our initial questionnaire development work with family-level data showed that typical good days’ catch data were less variable among fishers than those based on their recollected average catches, and would thus have greater power to detect changes [59, 65].

#### Socio-economic drivers of finfish depletions

A total of 423 fishers targeting at least one of the most vulnerable finfish species identified in models were interviewed in three of the marine KBAs (Lanuza Bay, Polillo and Honda Bay) to gather data on potential socio-economic influences on fishery depletions. The socio-economic data included overall daily income, overall daily savings, number of people in the household, number of children, community population size, area of delineated fishing ground, reef area per marine KBA, distance of market from community, hours per day fishing, engine power and community development. To assess community development, we used a composite index based on the presence of community-level infrastructure and services [66, 67] including primary school, secondary school, hard top road, mobile phone signal, variety store, electricity supply, piped water system, septic tank, regular jeepney trips, regular bus trips, fuel service, sewage treatment facility and health care centers.

### Statistical analysis

#### Perceived CPUE temporal trends

Finfish species for detailed temporal analysis were selected based on their reported catch occurrence in all five areas and their frequency of zero reported catches. We evaluated different statistical models for each of these species to estimate the change in perceived CPUE in relation to time and fishing practices (Tables 1 and 2). We tested for the best statistical approach for each species that would reduce the overall variation in the data [68] using Generalized Linear Mixed Models (GLMMs) to incorporate any random component that estimates the heterogeneity between clusters (i.e. between subjects) [69] and zero-inflated GLMMs to account for excessive zeroes in the perceived CPUE data.

**Table 1:** Response variable, fixed and random effects, dispersion and AIC values of the GLMM and zero-inflated GLMM (ZIGLMM) models for *Alectis ciliaris*, *Cheilinus undulatus* and *Lutjanus argentimaculatus*.

Variables	Model 1 GLMM	Model 2 ZIGLMM	Model 3a GLMM	Model 4a GLMM	Model 5a GLMM	Model 6a GLMM	Model 7a GLMM	Model 8a GLMM
**Response variable**								
Log (CPUE+1)	X	X	X	X	X	X	X	X
**Fixed effects**								
Decadal year	X	X	X	X	X	X	X	X
Decadal age	X	X						
Decadal age^2^	X	X						
Main gear	X	X						
Engine power	X	X	X	X	X	X	X	X
Hours fishing	X	X						
Fishing experience	X	X	X	X	X	X	X	X
Interaction (decadal year x decadal age)							X	
Interaction (decadal year x engine power)								X
**Random effects**								
Interviewee	X	X	X	X	X	X	X	X
Decadal age				X		X	X	X
marine KBA					X	X	X	X
**Model criteria**	Dispersion/ AIC	Dispersion/ AIC	Dispersion/ AIC	Dispersion/ AIC	Dispersion/ AIC	Dispersion/ AIC	Dispersion/ AIC	Dispersion/ AIC
*Alectis ciliaris*	0/7201	0/9332	0/7161	0/6877	0/7102	0/6799*	0/6805	0/6811
*Cheilinus undulatus*	0/3533	0/3735	0/3501	0/3315	0/3451	0/3251*	0/3280	0/3262
*Lutjanus argentimaculatus*	0/6931	0/9410	0/6890	0/6517	0/6772	0/6396*	0/6402	0/6409

X = explanatory variable used in the model;

* = selected model

**Table 2:** Response variable, fixed and random effects, dispersion and AIC values of the GLMM and zero-inflated GLMM (ZIGLMM) models for *Bolbometopon muricatum* and *Epinephelus lanceolatus*.

Variables	Model 1GLMM	Model 2 ZIGLMM	Model 3b ZI GLMM^a^	Model 4b ZI GLMM^b^	Model5b ZI GLMM^b^	Model 6b ZI GLMM^b^	Model 7b ZI GLMM^c^	Model 8b ZI GLMM^c^
**Response variable**								
log(CPUE+1)	X	X	X	X	X	X	X	X
**Fixed effects**								
Decadal year	X	X	X	X	X	X	X	X
Decadal age	X	X	X	X	X	X	X	X
Decadal age^2^	X	X	X	X	X	X	X	X
Main gear	X	X						
Engine power	X	X	X	X	X	X	X	X
Hours fishing	X	X						
Fishing experience	X	X	X	X	X	X	X	X
Interaction (decadal year x decadal age)							X	
Interaction (decadal year x engine power)								X
**Random effects**								
Interviewee	X	X	X	X	X	X	X	X
Decadal Age				X		X		
marine KBA					X	X	X	X
**Model criteria**	Dispersion/AIC	DispersionAIC	Dispersion//AIC	Dispersion/AIC	Dispersion/AIC	Dispersion/AIC	Dispersion//AIC	Dispersion/AIC
*Bolbometopon muricatum*	0/5553	0/5013	0/5034	0/5297	0/4879	0/4881	0/4848*	0/4879
*Epinephelus lanceolatus*	0/2356	0/2208	0/2199	0/2339	0/2185*	0/2187	0/ 2173	0/ 2186

X = explanatory variable used in the model;

* = selected model

^a^ Engine power and years of experience not significant as explanatory variable for *E*. *lanceolatus* (p>0.05)

^b^ Only decadal year is a significant explanatory variable for *E*. *lanceolatus* (p<0.05)

^c^ Decadal year, decadal age, decadal age^2^, engine power, fishing experience and interaction terms (Decadal year x decadal age; decadal year x engine power) are not significant explanatory variables for *E*. *lanceolatus* (p>0.05)

Seven explanatory variables were included in these statistical models: decadal year (i.e. 1950s, 1960s, 1970s, 1980s, 1990s, 2000s, current year at time of interview), main fishing gear, engine power in horsepower, number of hours fishing and number of years of fishing experience, decadal age (age at the midpoint of each decade), and decadal age squared, the last to test for any non-linear relationship between age of fisher and perceived CPUE.

Dispersion and Akaike Information Criterion (AIC) were used for model selection. Overdispersed models (Dispersion > 1) may be due to the non-normal distribution of the data (positively skewed because of the presence of some large catches per day), and perceived CPUE was logarithmically transformed to help normalize the data. In the GLMMs and zero-inflated GLMMs, the overall variability was separated into fixed and random components, the former estimating the effect of interest (e.g. of time and fishing practice on perceived CPUE), while the random component estimated the heterogeneity between interviewees [67].

Whether using GLMMs (Table 1) or zero-inflated GLMMs (Table 2), a stepwise approach was employed for each species to assess the significance of different random components by increasing the number of random effects in each step [68, 70]. In Models 3a and 3b (Tables 1 and 2), only the interviewee was treated as a random effect, accounting for individual differences among fishers. Models 4a and 4b (Tables 1 and 2) included both interviewee and decadal age. Models 5a and 5b (Tables 1 and 2) included interviewee nested in marine KBA, accounting for perceptions varying among marine KBAs. Models 6a and 6b (Tables 1 and 2) included decadal age and interviewee nested in marine KBA. The model with the lowest AIC was considered the best fit to the CPUE data for each species that disappeared from catches while accounting for any fishing practice significantly affecting the data.

Interactions among explanatory variables were also tested: decadal year x decadal age for the relationship of decadal CPUE to age (Tables 1 and 2, Models 7a and 7b), and decadal year x engine power for the relationship of decadal CPUE to engine power (Models 8a and 8b).

To estimate the rate of change of perceived CPUE (percent decline) from the 1950s to 2012–2014 for each species, we also ran the selected final model including decadal year as a categorical variable. This allowed estimates of perceived CPUE in each decade to be estimated.

#### Life-history and socio-economic predictors of species disappearance

We used linear modelling to investigate relationships between species disappearance and life-history traits of the finfish species reported by fishers to be missing from catches. Where data were overdispersed we used negative binomial models to quantify aggregate. Mixed models with interviewee as the random effect were used to account for unmeasured variation among interviewees.

The modeled life-history traits were maximum total length [L_max_], the growth coefficient [*k*], maximum age at maturity [T_mat_], trophic level, and vulnerability coefficient based on Fishbase [57]) (Table 3). The relative importance of each explanatory variable was assessed based on its p-value. Models with the lowest AIC values were considered the best fit to the given data [68, 70], and model fit was validated by assessment of the residuals against the fitted values (S1 Fig).

**Table 3:** Response variables, fixed and random effects, dispersion and AIC values of GLM and GLMM models for the life-history traits analysis.

Variables	Model 1 Poisson GLM	Model 2 Negative Binomial GLM	Model 3 GLMM
**Response variable**			
log(CPUE+1)	X	X	X
**Fixed effects**			
Decadal year	X	X	X
Maximum length (L_max_)	X	X	X
Growth coefficient (*k*)	X	X	X
Age at first maturity (T_mat_)	X	X	X
Trophic level	X	X	X
Vulnerability coefficient	X	X	X
**Random effect**			
Interviewee	X	X	X
**Model criteria (Dispersion/AIC)**	130/Inf	10/93306	0/40173*

X = explanatory variable was included in the model; Inf = positive infinity;

* = selected model

A multivariate approach was used to assess any association of socio-economic variables (overall daily savings, overall daily income, number of household members, number of children, population of community, area of delineated fishing ground, reef area, distance of market from the community, hours per day fishing, engine power and a community development score) with perceived CPUE trends in the five most vulnerable finfish species in Lanuza Bay, Polillo and Honda Bay. For each species, the random effects of the final models of perceived CPUE decline were extracted. The relationship of the interviewee random intercepts for all five species were correlated with socio-economic drivers using Redundancy Analysis (RDA) [70]. A permutation test using the step function in the vegan package [71] including all the socio-economic drivers determined the significant drivers (p <0.05) which were then included in a second model. The model with the lower AIC was considered to best explain effects of the socio-economic variables.

GLM models were fitted using the MASS package [72], GLMM models used the nlme package [73] and ZIGLMMs were fitted using the glmmADMB package [74, 75] in R version 15.1 [76]. Model fit was validated by assessment of the residuals against the fitted values. Error plots were produced [70] using the ggplot2 package [77].

## Results

A total of 2655 fishers were interviewed in the five marine KBAs: 411(26% of total registered fishers) in Lanuza Bay, 955 (28%) in Danajon Bank, 455 (27%) in Verde Island Passage, 422 (19%) in Honda Bay and 405 (total number of registered fishers, unknown) in Polillo Islands (S1 Table). Seven fishers were excluded from the analysis because they did not target finfish, and a total of 1,830 respondents (69% of total), mostly in their mid and late life stages (S2 Fig) could answer questions about species that had disappeared from catches: 212 (11.6% of total) in Lanuza Bay, 600 (32.8%) in Danajon Bank, 364 (19.9%) in Verde Island Passage, 345(18.8%) in Polillo Islands and 309 (16.9%) in Honda Bay.

**Fig 2:**
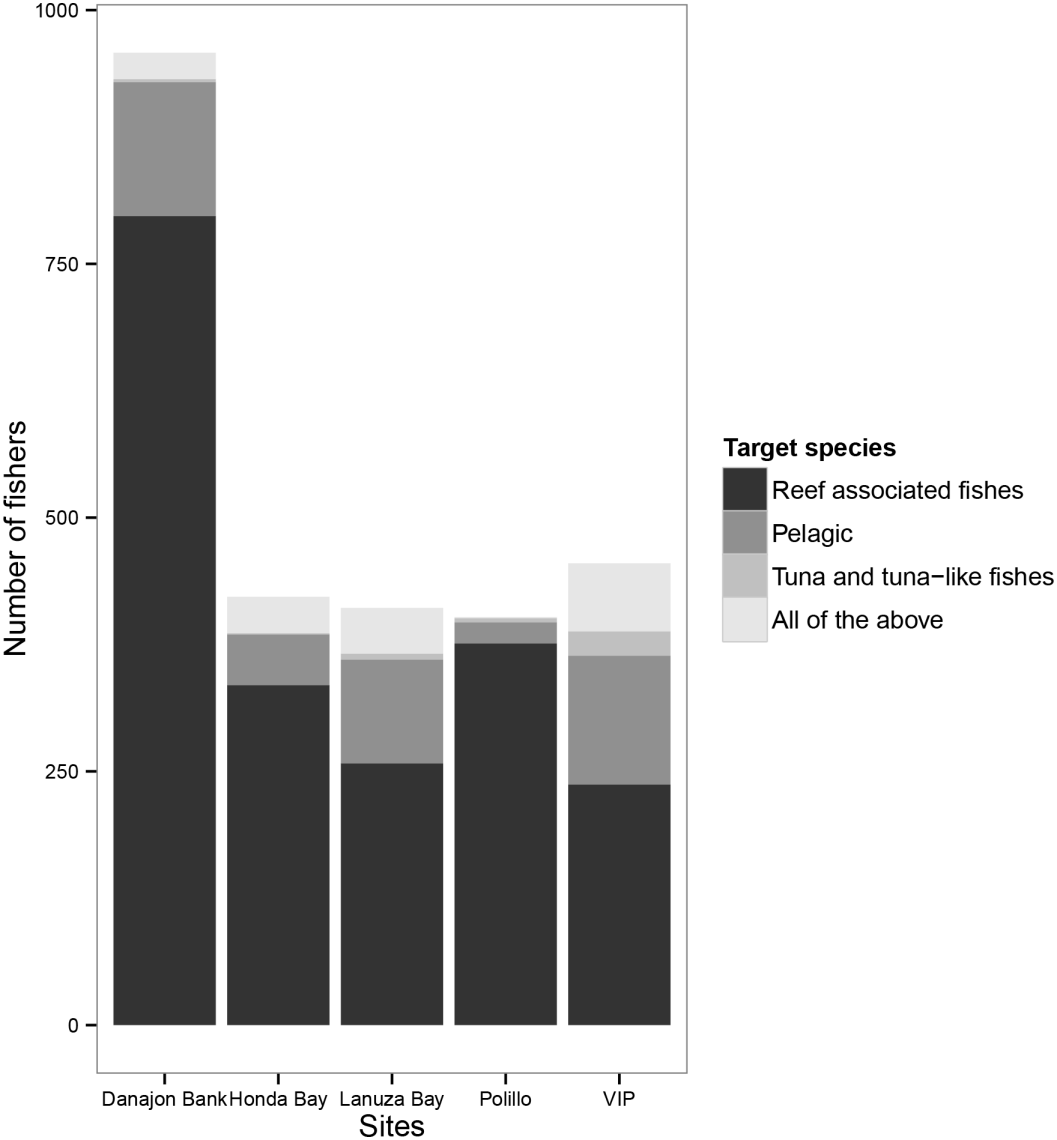
Target species of fishers in each marine KBA.

The distribution of the type of target species of the fishers is shown in (Fig 2) with two thousand and three fishers (76%) targeting reef-associated species. Fishery details including fishing gears, target species, other fishing practices and changes that have occurred over time in the five marine KBAs are included in Supplementary Information (S5 and S6 Tables) (S3, S4 and S5 Figs).

### Finfish disappearances from catches

A total of 59 finfish names were reported to have gone missing from catches between the 1950s and 2014, of which 42 (71%) were identified to species level, two (3%) to genus, seven (12%) to family and eight (14%) to local name only (S3 Table).

The five species with the highest frequency of zero reported catches in all five areas, which were considered the most vulnerable to depletion were the green bumphead parrotfish (n = 488 interviewees), African pompano (n = 1049), mangrove red snapper (n = 1065), humphead wrasse (n = 456) and giant grouper (n = 199) (Table 4). Based on dispersion and AIC model selection criteria, either GLMMs or ZIGLMMs most reduced the overall variability in the perceived CPUE.

**Table 4:** Numbers of zero catch reports for species reported in at least two marine KBAs.

Common English name	Species Name	Family	A	B	C	D	E	Total zero catch reports	N
Green bumphead parrotfish^a^	*Bolbometopon muricatum*	Labridae	82	111	24	48	36	301	488
African pompano^a^	*Alectis ciliaris*	Carangidae	151	24	8	29	51	263	1049
Mangrove red snapper^a^	*Lutjanus argentimaculatus*	Lutjanidae	148	20	18	28	40	254	1065
Humphead wrasse^a^	*Cheilinus undulatus*	Labridae	86	28	12	41	29	196	456
Giant grouper^a^	*Epinephelus lanceolatus*	Serranidae	19	21	11	21	16	88	199
Smalltooth emperor	*Lethrinus microdon*	Lethrinidae	32	0	12	23	26	93	723
Golden trevally	*Gnathanodon speciosus*	Carangidae	5	0	9	23	27	64	471
Leopard coral grouper	*Plectropomus leopardus*	Serranidae	3	12	0	2	0	17	19
Humpback grouper	*Cromileptes altivelis*	Serrranidae	1	0	1	11	0	13	17
Bicolor goatfish	*Parupeneus barberinoides*	Mullidae	1	2	1	0	0	4	13
Orange-spotted grouper	*Epinephelus coioides*	Serranidae	7	0	0	0	1	8	12
Oxeye scad	*Selar boops*	Carangidae	2	0	0	0	2	4	7
Blue trevally	*Carangoides ferdau*	Carangidae	0	1	0	0	2	3	3
Big eye trevally	*Caranx sexfasciatus*	Carangidae	1	0	0	1	0	2	2
Goldlined spinefoot	*Siganus guttatus*	Siganidae	1	1	0	0	0	2	3

A = Danajon Bank, B = Lanuza Bay, C = Honda Bay, D = Polillo Islands, E = VIP, N = total number of respondents

^a^Species selected for detailed temporal analysis

GLMMs for humphead wrasse, African pompano and red mangrove snapper showed only decadal year, engine power and years’ fishing experience accounted for significant variation in perceived CPUE (Table 1, Model 1). Including only these covariates (Table 1, Model 3a) reduced the AIC. Including interviewee, marine KBA and fisher decadal age as random effects further improved the AIC (Table 1, Model 6a) while adding interaction terms did not. The final model for these species shows both decadal year and fishing experience negatively correlated, while engine power was positively correlated, with perceived CPUE (Table 5).

**Table 5:** Covariates included in the final model (see text) for each species showing statistical significance and direction of effect (+/-).

Covariates	*Alectis ciliaris* (n = 1049)	*Bolbometopon muricatum* (n = 488)	*Cheilinus undulatus* (n = 456)	*Epinephelus lanceolatus* (n = 199)	*Lutjanus argentimaculatus* (n = 1065)
Decadal year	***(-)	***(-)	***(-)	***(-)	***(-)
Decadal age		**(+)			
Decadal age^2^		**(-)			
Gear					
Engine power	***(+)	** (+)	* (+)		**(+)
Hours fishing					
Fishing experience	**(-)	***(-)	* (-)		**(-)
Interaction (decadalyear x Decadal age)		**(-)			
Interaction (decadalyear x engine power)					
Perceived CPUE decline	66%	88%	82%	74%	64%

* = p<0.05,

** = p<0.01,

*** = p<0.001

Decadal year, decadal age, decadal age^2^, engine power and years’ fishing experience explained significant variation in the perceived CPUE data for green bumphead parrotfish and giant grouper (Table 2, Model 3b). Inclusion of marine KBA and interviewee as random effects improved the AIC further (Table 2, Model 5b) with decadal year the only significant explanatory variable for the giant grouper. Adding the interaction decadal year x decadal age with interviewee and marine KBA as random components further reduced AIC in both species (Table 2, Model 7b).

This model for green bumphead parrotfish (Table 2, Model 7b) showed decadal year, years’ fishing experience and the interaction between decadal year and decadal age negatively correlated with perceived CPUE while engine power was positively correlated with perceived CPUE (Table 5). There was also a significant quadratic relationship between decadal age and perceived CPUE (Table 5).

For the giant grouper, decadal year and the interaction between decadal year and decadal age were not significant (Table 2, Model 7b), thus Model 5b (Table 2) was selected as the final model, with decadal year alone significantly describing the decline of perceived CPUE through time.

**Fig 3:**
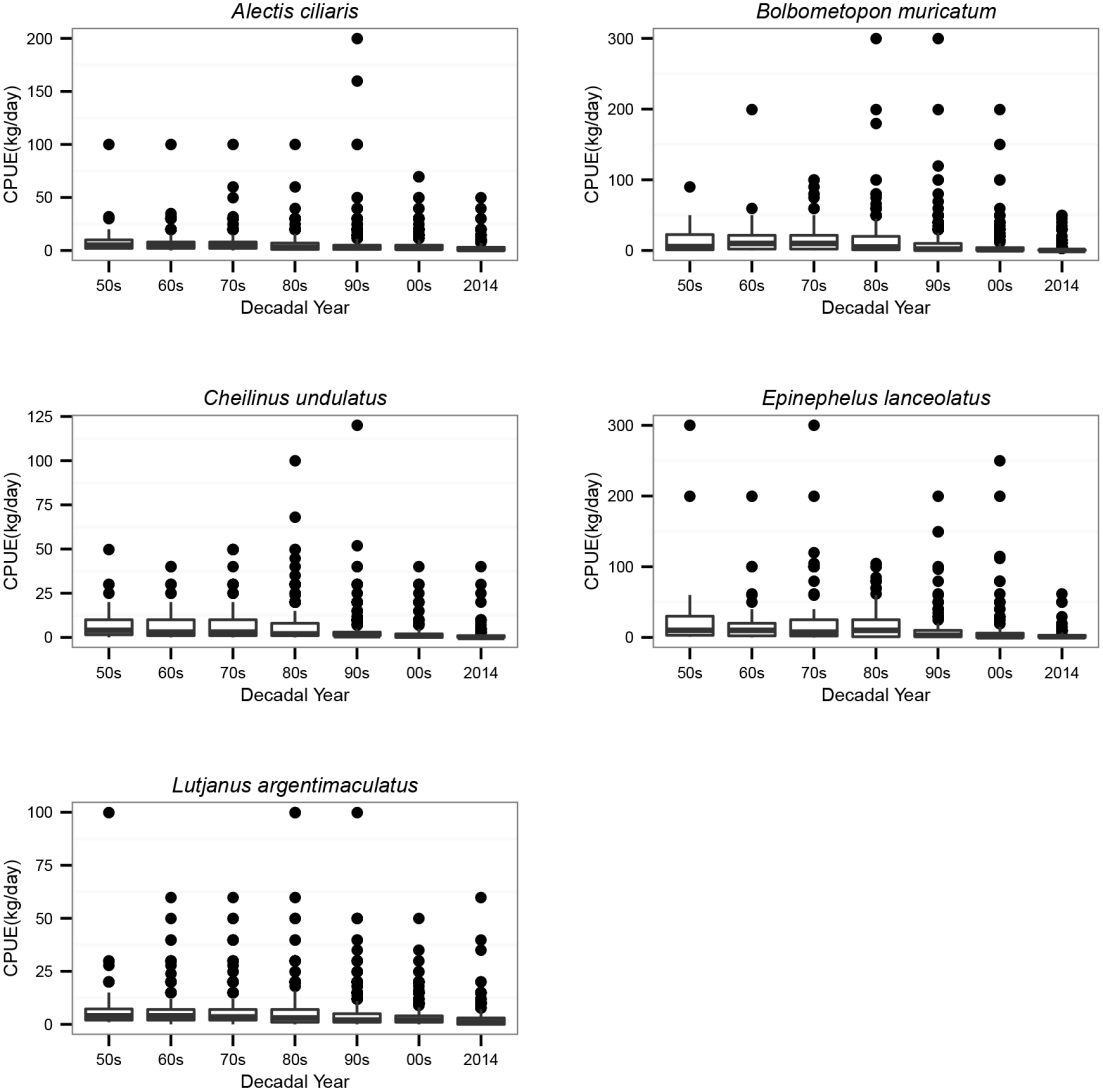
Boxplots of catch per unit effort (kg/day) for the five key species. Perceived CPUE outliers for some species were removed: *E*. *lanceolatus* (576 kg/day); *B*. *muricatum* (600 kg/day) and *A*. *ciliaris* (700 kg/day).

**Fig 4:**
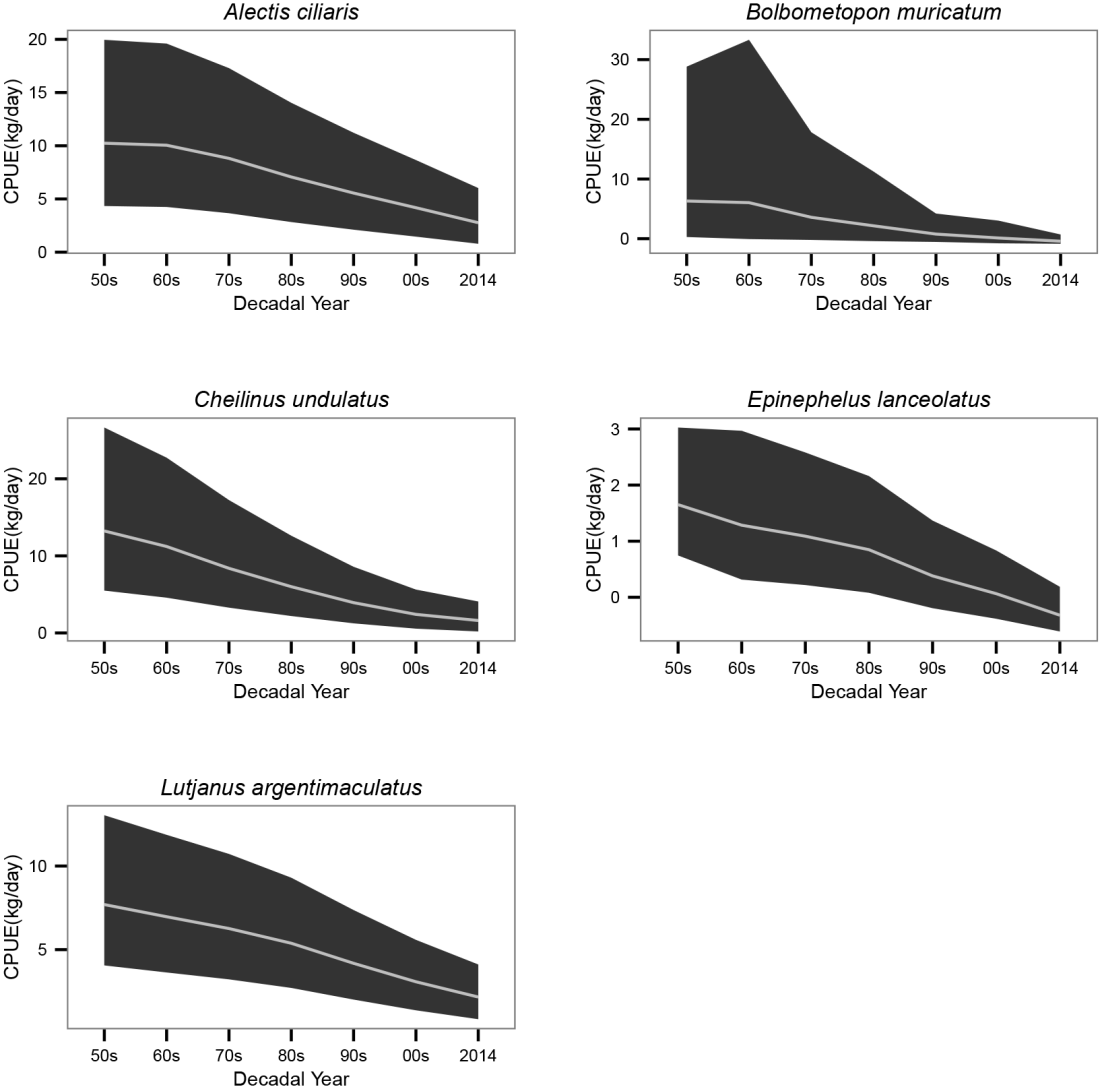
Perceived Catch per unit effort (CPUE) ± SE.

Recalled good days’ catches of the 5 selected species tended to decline across all the decades (Fig 3). The final models indicated that perceived CPUE declined between the 1950s and 2014 by 82% in the humphead wrasse, 66% in African pompano, 64% in mangrove red snapper, 88% in green bumphead parrotfish and 74% in giant grouper (Table 5, Fig 4).

#### Life-history traits as predictors of disappearance from catch

Of the life-history variables (S4 Table), the selected models showed decadal year and species’ maximum lengths negatively correlated, and the *k* growth coefficient, age at first maturity, trophic level and vulnerability coefficient positively correlated, with the perceived CPUE of the 42 species that had disappeared from catches (Table 6).

**Table 6:** Life-history covariates included in the final model showing statistical significance and direction of effect (+/-).

Covariates	*p*-value
Decadal year	***(-)
L_max_	***(-)
*k*	***(+)
T_mat_	***(+)
Trophic level	**(+)
Vulnerability coefficient	**(+)

* = p<0.05,

** = p<0.01,

*** = p<0.001

#### Socio-economic factors of finfish depletions

**Fig 5:**
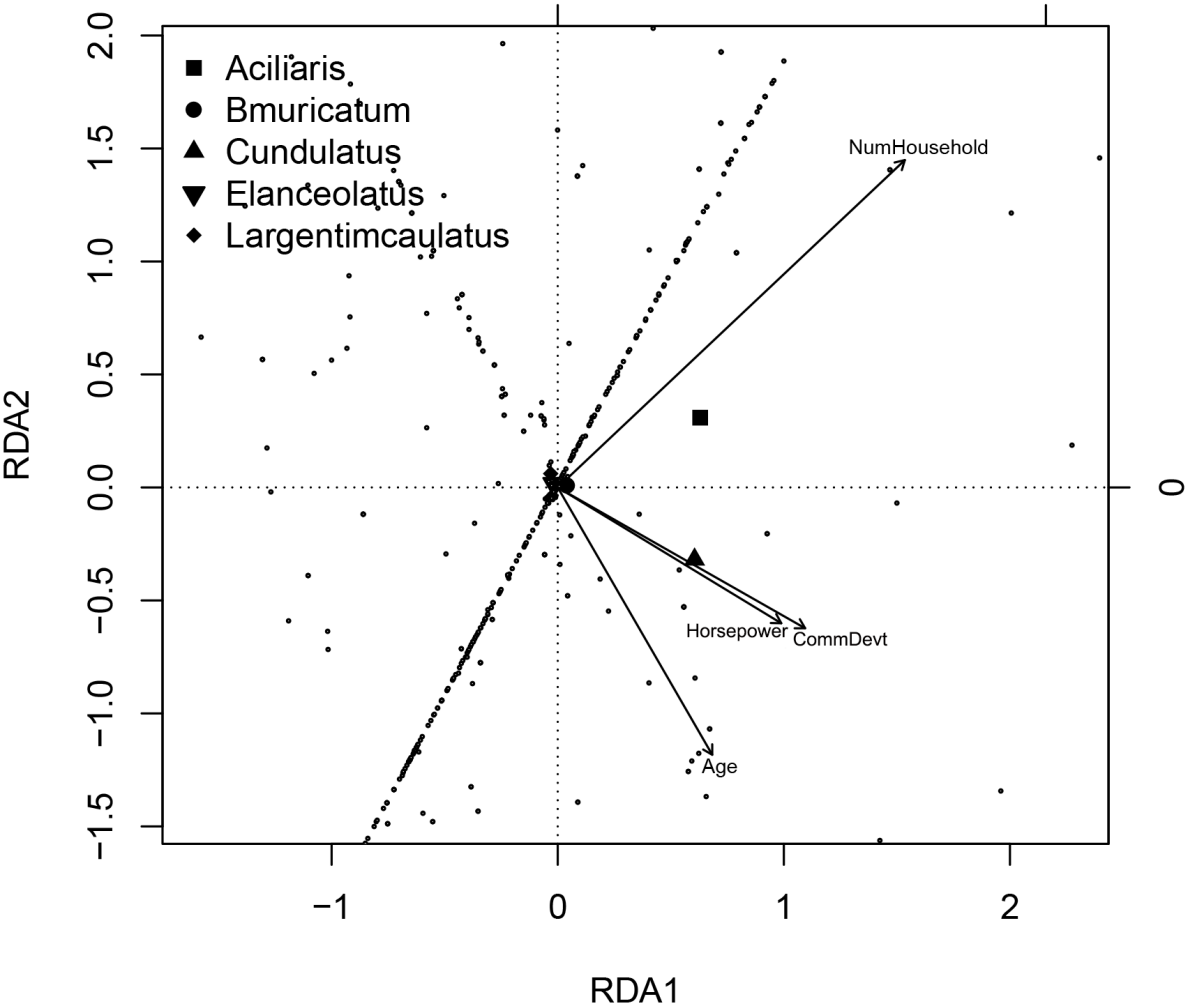
RDA plot of species and socio-economic drivers based on Model B.

Fisher age, number of household members, engine power and community development index were significant explanatory socio-economic variables associated with depletion of finfish species based on the stepwise permutation test. The model including only these significant socio-economic variables had a lower AIC (-98) compared to that containing all the socio-economic variables (Table 7). A redundancy analysis of the better model showed that 3.8% of the variation in rate of change of perceived CPUE among target species was attributable to fisher age, number of household members, engine power and community development index. Among the five most vulnerable species in the three areas (Lanuza Bay, Polillo and Honda Bay), the decline of *Cheilinus undulatus* was most closely influenced by fisher age, engine power and community development index, while that of *Alectis ciliaris* was closely related with the number of household members (Fig 5).

**Table 7:** Candidate models for the socio-economic variables depletion.

Model	Model Parameters	AIC
Model A	species ~ age + overall savings + overall income + No. of household members + No. of children + community population + area of delineated fishing ground + distance of market from community + hours fishing + engine power + Community Development Index + reef area	-83
Model B	species ~ age + No. of household members + engine power + Community Development Index	-91

## Discussion

We identified 59 finfish names disappearing from catches at various points since the 1950s. The five most vulnerable species (*B*. *muricatum*, *E*. *lanceolatus*, *C*. *undulatus*, *A*. *ciliaris* and *L*. *argentimaculatus*), those with the highest frequency of zero catches, exhibited 64–88% declines in perceived good days’ CPUE. These disappearances from catches were mainly associated with excess and uncontrolled fishing, fish life-history traits like large maximum body size and some indirect socio-economic drivers like access to market-related community level infrastructures and services and human population.

Among the five most vulnerable species, the green bumphead parrotfish *B*. *muricatum* had the greatest proportion of fishers reporting no catches, the most fishers targeting it and the greatest decline in perceived CPUE. Extensive underwater visual surveys show that this species is very rare in the Philippines [22]. *B*. *muricatum* has also been heavily fished and is rare in most of its Indo-Pacific range [22, 24, 78, 79]. This species is considered Vulnerable in the IUCN Red List [77], while. *C*. *undulatus* is categorized as Endangered and is in the Convention on International Trade of Endangered Species of Wild Flora and Fauna (CITES) Appendix II since 2004 [80]. Its rarity and vulnerability [57] have been highlighted and its densities rarely exceed 20 fish per hectare in preferred outer reef habitat [81]. Of the five most vulnerable species, *E*. *lanceolatus* and C. *undulatus* are the most targeted for the Asian live reef food fish trade [81, 82]. There is now extensive hatchery production of *E*. *lanceolatus*, however large individuals for brood stock and consumption remain wild caught [83], sourced, among others, from Indonesia and the Philippines [84]. Juveniles of *C*. *undulatus* are preferred by consumers and fetch the highest prices in this trade [85]. Despite regulations for *C*. *undulatus* in the Philippines, there is much illegal, unregulated and unmonitored fishing [85] and in our study, many fishers were unaware that it is legally protected. *A*. *ciliaris* is categorized by IUCN as Least Concern [86], but it is extinct at two islands off Bohol (Philippines) [27], while *L*. *argentimaculatus* remains unassessed by IUCN (2015) yet it may have been extirpated in Naujan Lake National Park in Mindoro Oriental (Philippines) [87].

### Potential biases in the perceived CPUE data

There are potential biases in catch data derived from fisher interviews [40, 60, 88], and changes in recollections may mask or exaggerate perceived trends [40, 60, 88, 89]. Older and more experienced fishers may be more likely to report greater decline or more species disappearing. The decadal time scale of recalled catches likely contributed to variability in the data [88]. Excessive zeroes in our data may have arisen for several reasons. Zeroes in the data can be classified as “false” or “true” [68], the “true” zero CPUE values occurring when a species is absent resulting in low probability of fishing success, while “false” zeroes occur when a target species is present but not caught, or forgotten, by the fisher interviewee. Thus a target species may be genuinely rare, or have low catchability with the fishing gear involved, or it might be a schooling fish where there is a high probability of zero catches but catch rate might occasionally be very high if it is encountered [90]. In this study, this was accounted for by using zero-inflated GLMM models. Increasing fishing efficiency through fishers’ greater fishing experience or engine power, which were significant in catch trends for deep dwelling species such as adult African pompano, mangrove red snapper and humphead wrasse, are among other potential biases in such temporal data [60, 88]. It is important to evaluate such biases in assessing extirpation risk, however a standard method to quantify such bias has yet to be developed [40]. For the species found here to be most vulnerable being mostly wide-ranging [57], comparable analyses from multiple data sources at wider spatial scales are desirable.

### Drivers of finfish depletion

That maximum body size and growth coefficient were among the best predictors of declines in species’ perceived CPUE corroborates much theoretical and empirical analysis [45–48, 91–97]. Large-bodied fishes are usually targeted most heavily due to their high value and catchability [47] but also tend to have greater ages at maturity, and low intrinsic rates of population increase [48, 96, 98], although this is not universally the case for reef fishes [44, 99]. Differences in fishing intensity and strategy including different fishing gears and techniques are also important factors in fish depletion [46, 95].

Fishing affects reef fishes directly through removal of individuals and indirectly by altering species interactions or habitat [39, 100–102]. The modelling indicated that the green bumphead parrotfish and giant grouper had been targeted and depleted the longest. Heavy exploitation of these species likely started before the 1950s, their being the largest species of their kind probably enhancing their desirability to fishers. A prominent feature of the modeled catch trend in green bumphead parrotfish was the quadratic relationship between perceived CPUE and decadal age. Fishers’ catches have tended to increase with age early in their fishing careers, reach a peak, and then decline with age. This pattern is attributable to the physical exertion required by spearfishing, which was the main reported method for targeting this species [27, 103]. Number of years’ fishing experience was negatively related with perceived CPUE for bumphead parrotfish, humphead wrasse, African pompano and mangrove red snapper. This may be fisher-behaviour and/or ecological in origin, for example fishers switching target species or fishing grounds as a stock is depleted. Perceived CPUE declines of the five most vulnerable species commencing in the 1960s and 1970s coincided with many changes in fishing practice. Poison fishing commenced in the 1960s, fine mesh nets, trawls and intensive but destructive fishing gears were introduced in the 1970s, and commercial fishers using sophisticated fishing gears and lights appeared in the 1990s [104]. Gillnets, once single-layered and only a few metres in length, were now 2–3 ply and up to 5000 m long. Hook and line developed from simple hand lines with few hooks into lines with up to 5000 hooks. In Honda Bay, a 40% increase in number of fishing boats between 1999 and 2002 likely contributed to reductions in CPUE and numbers of finfish species caught [105]. The Philippines’ multi-species fisheries remain exceptionally diverse in the gears used and this represents a huge challenge to attempt to quantify fishing effort [106].

We found that extending the time spent fishing was a common strategy to compensate for declining catches. Maximum fishing time had increased four-fold from the 1950s until 2014. Moving further away from the shore to maintain catches involves greater engine power and fuel costs. Our models show that the perceived CPUE was positively related to engine power in the humphead wrasse, mangrove red snapper, African pompano and green bumphead parrotfish. Given the distribution of these species in deeper waters [23, 57, 60, 62, 107, 108], more powerful engines are needed to reach these fishing grounds. The green bumphead parrotfish occurs in shallow waters, but its presence is increasingly rare there. In our models, despite greater fishing experience and engine power, catches have continued to dwindle. Fishers have been driven to use more efficient but destructive fishing methods [109]. In the Polillo Islands, fisheries law enforcement is insufficient to deter dynamite use and other illegal forms of fishing. Much of the exploitation is opportunistic, including the incidental catching of rare desirable species while targeting less desirable common species [110].

Social and economic factors are also important for tailoring management measures of coral reef fisheries [50]. Our models revealed that socio-economic factors such as fisher age, engine power as a proxy measure of wealth, and community development, may indirectly enhance depletion of *C*. *undulatus* and *A*. *ciliaris* in Lanuza Bay, Polillo Islands and Honda Bay. Juvenile humphead wrasses were mainly caught by cyanide and compressor fishing. As this is a form of fishing more suited to younger fishers, it may explain the role of fisher age in fishing success. This species is also a major target for the live reef food fish trade in which it commands among the highest prices. The capacity to generate greater income may explain why fishers from larger households tend to target this species. *C*. *undulatus* occurs mostly in deeper and more offshore reefs [57] and the relationship between perceived CPUE and engine power is linked to the ability of fishers to purchase more powerful boats and fuel to access such sites. Declines of humphead wrasse were also related to market-related infrastructure such as availability of mobile phone signal and regular transport, fuel supply and road surfacing. Market promixity can negatively affect vulnerable reef fish [50], while road construction has increased market access and thus fish sales, thereby increasing pressure on fish stocks [111]. Number of household members was also evidently conducive to depletion of *A*. *ciliaris*. The tendency for number of household members to enhance trends in declines of finfish highlights how increasing population size is putting more pressure on natural resources than in the past.

### Implications for conservation and management

The depletion of wide-ranging, once common schooling reef fish such as the green bumphead parrotfish is now widespread [24]. This species plays important ecological roles in coral predation and erosion [78, 112–115]. The declining abundance of this and related scraping species has likely already significantly impacted ecosystem functionality in several ways, including supply of carbonate sediment [79], suppression of macroalgae and maintenance of coral cover [115–120]. Our documenting of the decline of these large species indicates that effective conservation action is badly needed [112–114].

Substantial depletion of humphead wrasse, giant grouper, African pompano and mangrove red snapper in the Philippine marine KBAs contrasts with their occurrence in the wild and in markets [57]. With wide Indo-Pacific distributions, these fishes will long persist at some sites; global extinction is expected to be slow [121]. Rather, the tendency to depletion of wide-ranging, large and slow-growing reef fish species has implications for area-based conservation and regional resource management. Marine protected areas (MPAs) need to be large or strategically located within a network to capture the full ranges of such species [83]. They may need to encompass nurserv habitats for species like *L*. *argentimaculatus* [107, 108] and the present study highlights the need to include outer and deep (>30m) shelf areas in MPA planning [83]. Our evidence points to a distinct need for species-specific data, especially in these multi-species multi-gear fisheries such as here where aggregated catch data risk masking losses of vulnerable species such as *E*. *lanceolatus* [83].

High Filipino population growth [122] with more than 50% people living near or at the coast [20] is putting huge pressure on reef resources [50, 100, 101, 123–125]. This poses a great challenge to conservation in the face of poverty, movement of goods and people, and globalization [50]. Without initiatives that address these pressures, our data indicate for this epicentre of reef fish diversity that species are likely be lost before they have been scientifically characterized [126–128] and their ecosystem roles have been understood [129]. It is possible that the eight local names given by fishers for fishes no longer caught that could not be found in field guides may be significant in this regard.

## Conclusions

This paper contributes to the growing evidence for dramatic declines of vulnerable reef fish species in a highly species-rich but data-depauperate setting [30, 35, 38, 88]. Fishers’ knowledge provides evidence of local extinction vulnerability of many finfish species [24, 28, 32, 33, 37, 79, 130–132] and the links of this to life-history traits [27, 44–49], overexploitation [27, 43, 44] and socio-economic drivers [50, 51] of depletions. Our robust modelling [31, 133–135] of these data is novel for this global epicentre of coastal species diversity and highlights the value of fishers’ knowledge in providing evidence for declines in vulnerable species in abundances at large spatial and temporal scales.

## Supporting Information

S1 Fig Comparison of fitted values versus standardized residuals between Poisson Generalized Linear Model (Poisson GLM), Negative binomial GLM and Generalized Linear Mixed Model (GLMM) obtained from the life history trait analysis. (TIF)

S2 Fig Box plot of total number of species reported per age category. Early:21-41years old; Mid:42–62 years old; Late≥63 years old
(TIF)

S3 Fig Maximum fishing time of fishers in each of the six decades and current time of interview. (TIF)

S4 Fig Reasons for change in fishing ground. (TIF)

S5 Fig Types of main fishing gear per marine KBA. (TIF)

S6 Fig Fitted values versus standardized residuals obtained from the final CPUE temporal trend models. Residuals show a pattern of bands given by the number of zeroes in the data, which is a characteristic to all linear regression, GLM, mixed models and GAM models when there are lots of observations with the same values. Values larger than 2 or -2 are potential outliers [70].
(TIF)

S7 Fig Normal qq plots obtained from the final models allowing the assumption of normality to be checked. The two key species, *C*. *undulatus*, and *E*. *lanceolatus*, show resulting points that lie roughly on a straight line, indicating the distribution of the data is considered to be the same as normally distributed variable, than *B*. *muricatum*, *A*.*ciliaris* and *L*. *argentimaculatus* [70].
(TIF)

S1 Table Site characteristics with numbers and age categories of fishers interviewed. Early: 21–41 years, Mid: 42–62 years, Late-aged: ≥63 years
(DOCX)

S2 Table Questions asked in interviews based on Lavides et al. 2010 [27]. (DOCX)

S3 Table List of species reported to be disappearing from catches. (DOCX)

S4 Table Life History traits of the finfish names identified up to the species level. L_max_ = maximum body size, T_mat_ = age at first maturity, *k* = growth coefficient.
(DOCX)

S5 Table Main fishing gears used by fishers per marine KBA. (DOCX)

S6 Table Target species of fishers in each marine KBA. (DOCX)

S7 Table Variables included in initial models used in GLMM Analysis. (DOCX)
