# Supplementary figures and images for: Patterns of Coral-Reef Finfish Species Disappearances Inferred from Fishers’ Knowledge in Global Epicentre of Marine Shorefish Diversity
Source: PLoS One. 2016 May 18;11(5):e0155752. doi: 10.1371/journal.pone.0155752 (PMC4871521; doi:10.1371/journal.pone.0155752)

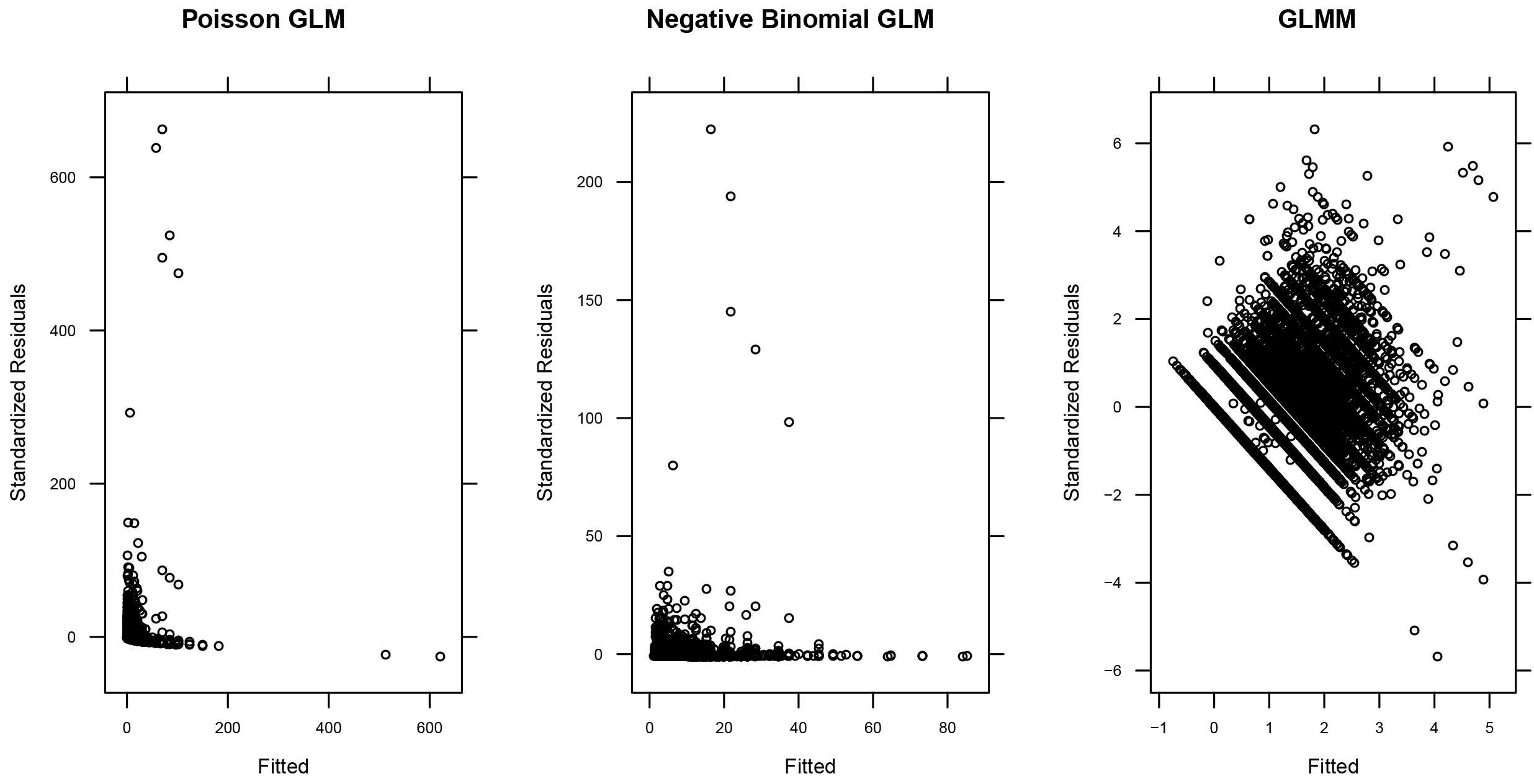

Supplement: S1 Fig — (TIF) [file pone.0155752.s001.tif]

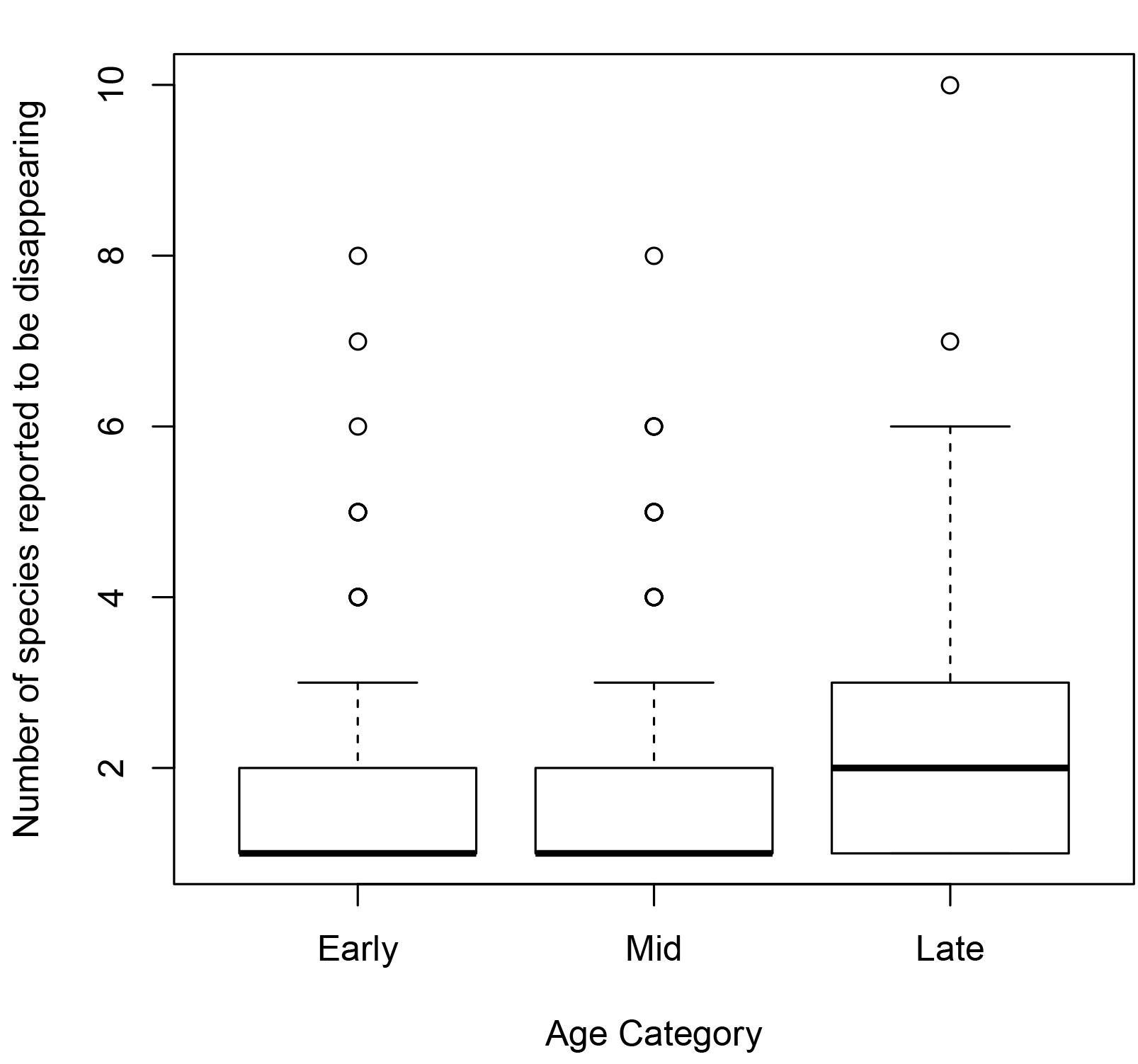

Supplement: S2 Fig — Early:21-41years old; Mid:42–62 years old; Late≥63 years old (TIF) [file pone.0155752.s002.tif]

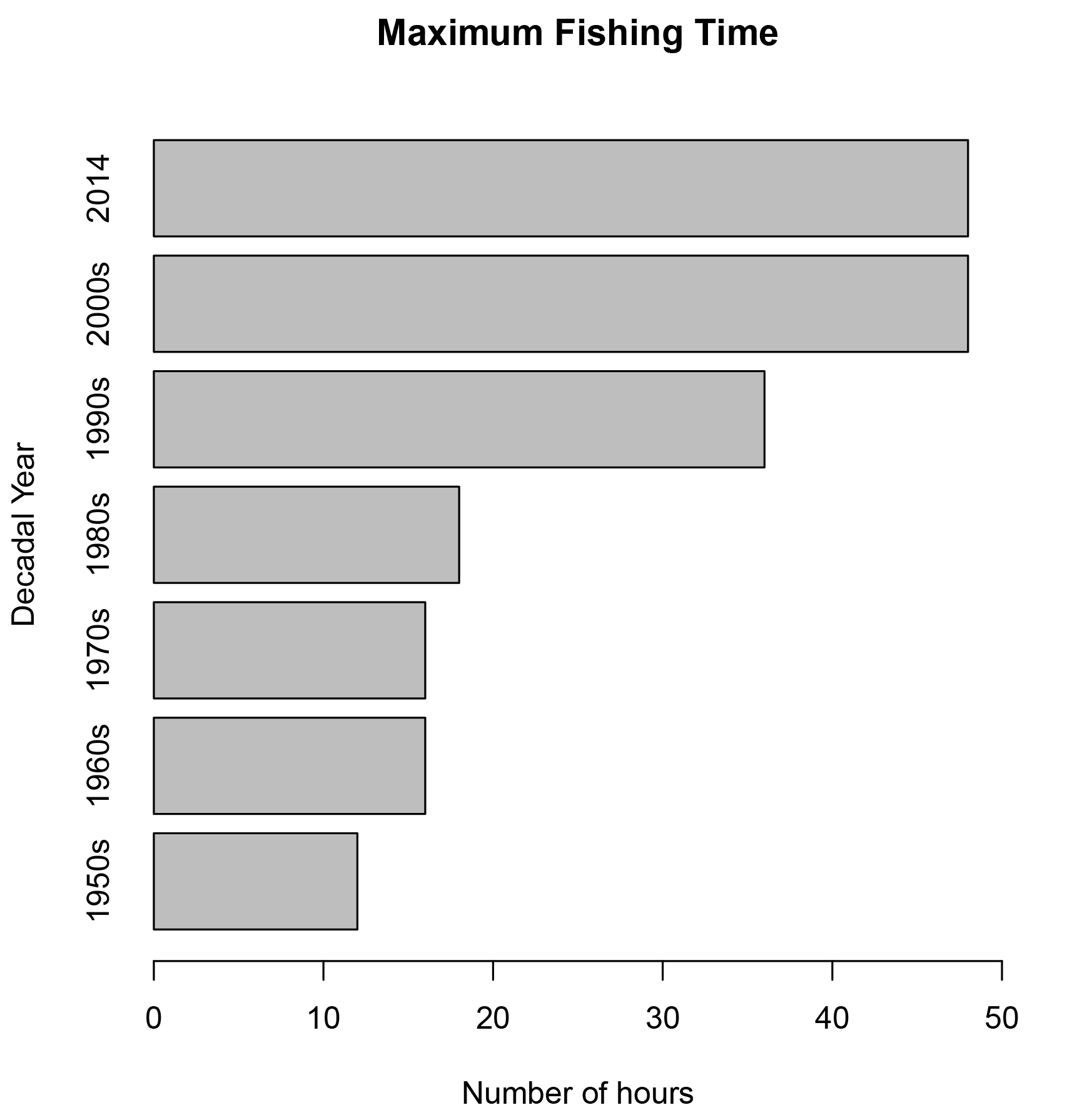

Supplement: S3 Fig — (TIF) [file pone.0155752.s003.tif]

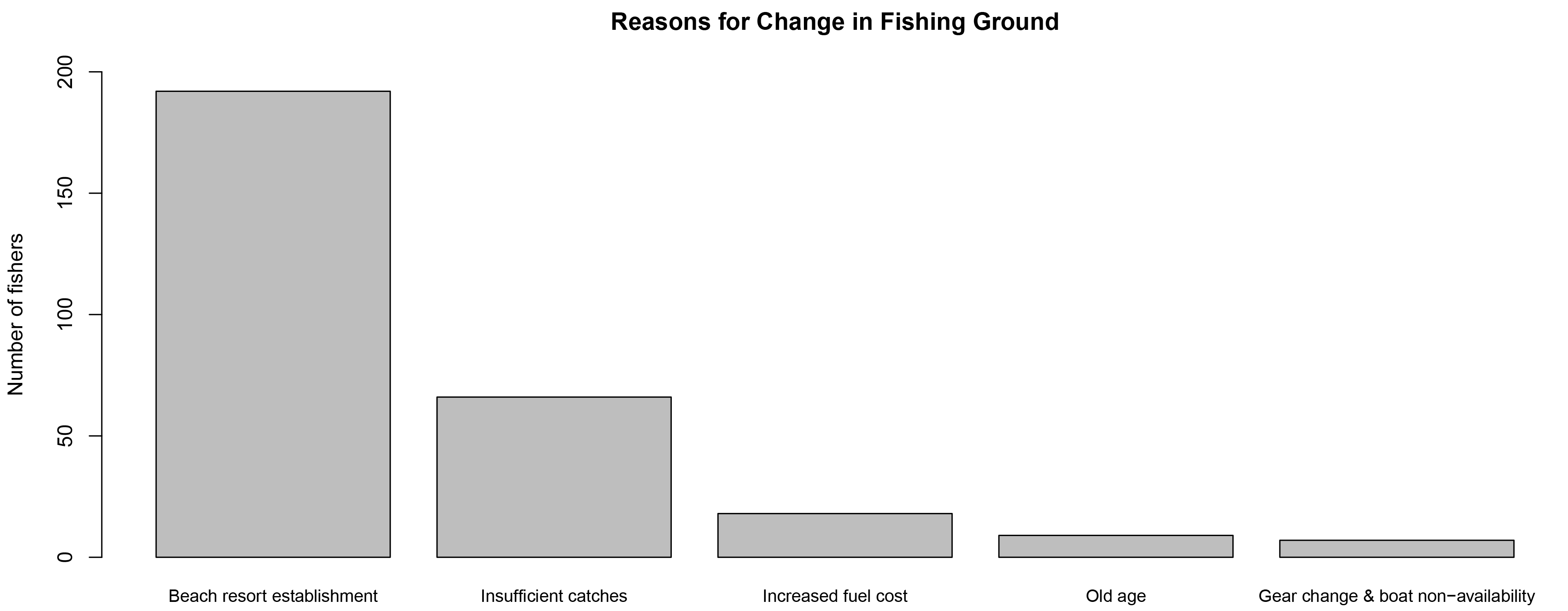

Supplement: S4 Fig — (TIF) [file pone.0155752.s004.tif]

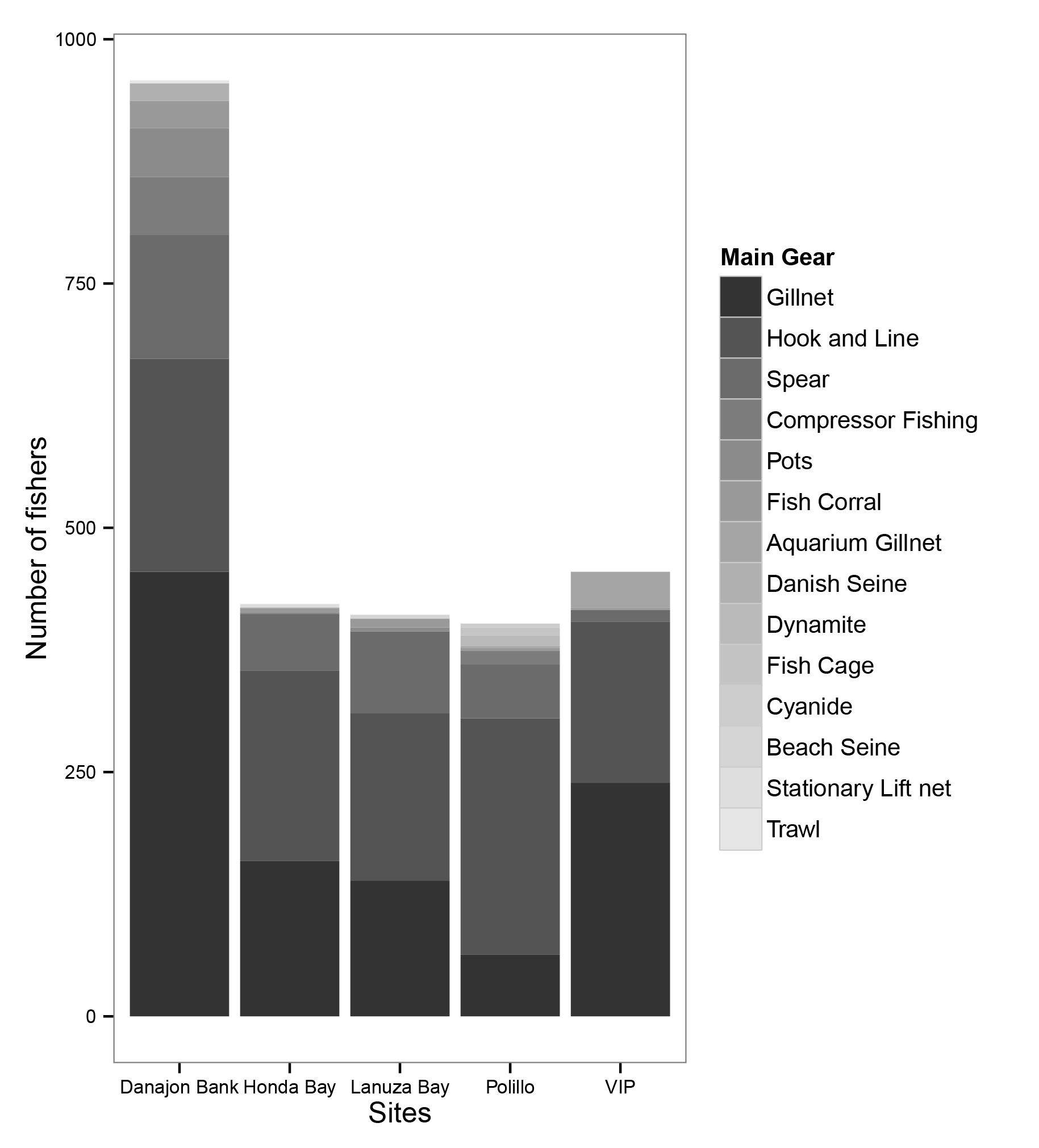

Supplement: S5 Fig — (TIF) [file pone.0155752.s005.tif]

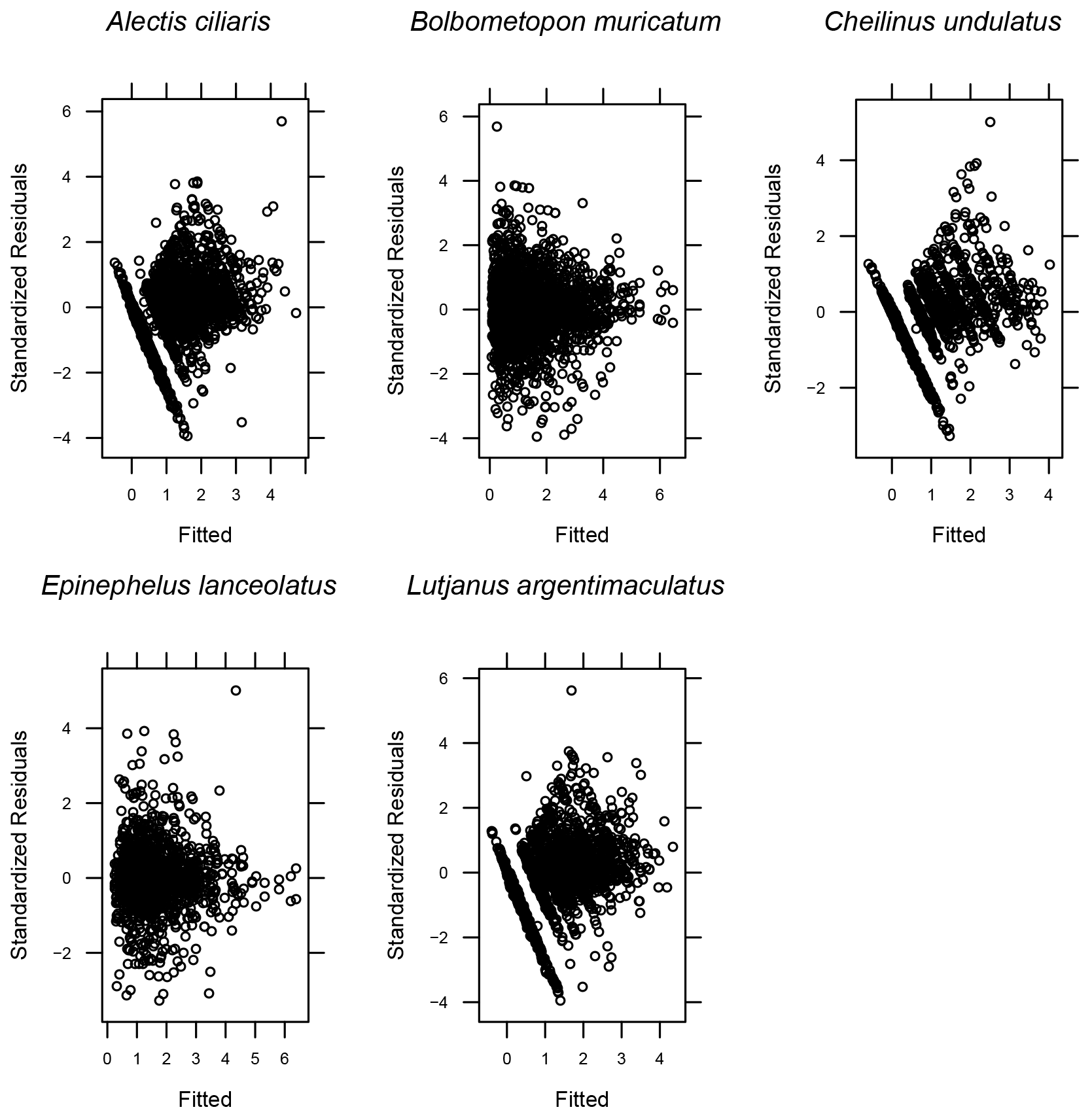

Supplement: S6 Fig — Residuals show a pattern of bands given by the number of zeroes in the data, which is a characteristic to all linear regression, GLM, mixed models and GAM models when there are lots of observations with the same values. Values larger than 2 or -2 are potential outliers [70]. (TIF) [file pone.0155752.s006.tif]

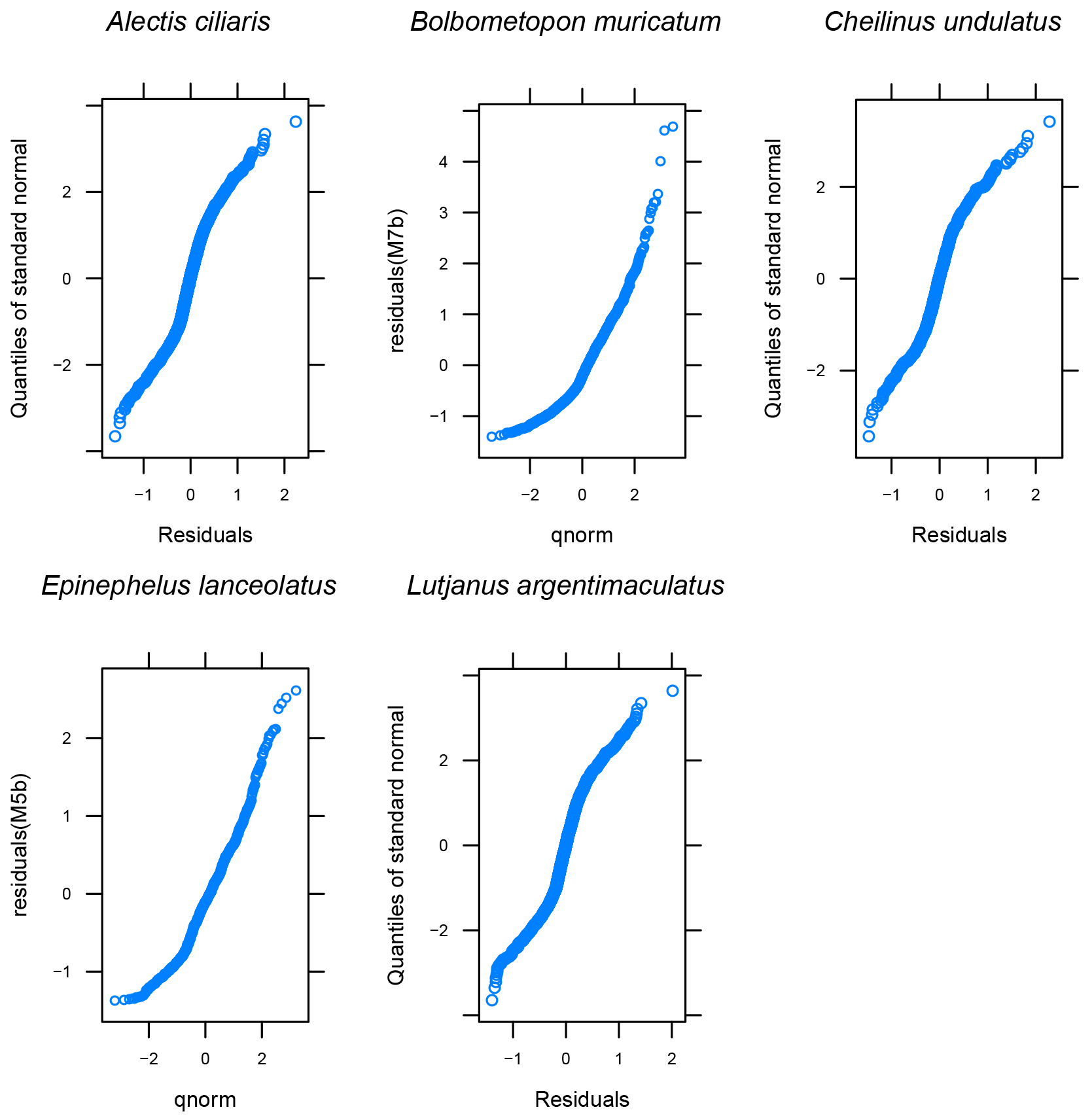

Supplement: S7 Fig — The two key species, C. undulatus, and E. lanceolatus, show resulting points that lie roughly on a straight line, indicating the distribution of the data is considered to be the same as normally distributed variable, than B. muricatum, A.ciliaris and L. argentimaculatus [70]. (TIF) [file pone.0155752.s007.tif]
