# Supplementary material for: Patterns of Coral-Reef Finfish Species Disappearances Inferred from Fishers’ Knowledge in Global Epicentre of Marine Shorefish Diversity
Source: PLoS One. 2016 May 18;11(5):e0155752. doi: 10.1371/journal.pone.0155752 (PMC4871521; doi:10.1371/journal.pone.0155752)
Supplement: S1 Table — Early: 21–41 years, Mid: 42–62 years, Late-aged: ≥63 years (DOCX) [file pone.0155752.s008.docx]

**Table S1.** **Site characteristics with numbers and age categories of fishers interviewed.**

| **Marine KBA** | **Sampling location** | **Population in 2010** | **Registered fishers**  **(No.)** | **Respondents’ age** | | | **Total respondents**  **(No.)** |
| --- | --- | --- | --- | --- | --- | --- | --- |
|  |  |  |  | **Early** | **Mid** | **Late** |  |
| **Lanuza Bay** | Lanuza | 11,857 | 240 | 32 | 42 | 24 | 98 |
|  | Cortes | 15,541 | 1200 | 111 | 145 | 57 | 313 |
| **Danajon Bank** | Bien Unido | 25,796 | 606 | 46 | 70 | 43 | 159 |
|  | Getafe | 27,788 | 686 | 84 | 70 | 21 | 175 |
|  | Talibon | 61,373 | 2176 | 267 | 251 | 103 | 621 |
| **Verde Island Passage** | San Juan | 94,291 | 1014 | 108 | 119 | 43 | 270 |
|  | Batangas City (Verde Island) | 5,491 | 130 | 25 | 26 | 9 | 60 |
|  | Lubang | 23,068 | 518 | 40 | 64 | 21 | 125 |
| **Polillo Islands** | Burdeos | 24,166 | - | 114 | 136 | 33 | 283 |
|  | Patnanungnan | 13,865 | - | 60 | 53 | 9 | 122 |
| **Honda Bay** | Honda Bay, Puerto Princesa | 92,304 | 2246 | 181 | 201 | 40 | 422 |

Early=21-41 years, Mid=42-62 years, Late ≥63 years
