# Supplementary material for: Patterns of Coral-Reef Finfish Species Disappearances Inferred from Fishers’ Knowledge in Global Epicentre of Marine Shorefish Diversity
Source: PLoS One. 2016 May 18;11(5):e0155752. doi: 10.1371/journal.pone.0155752 (PMC4871521; doi:10.1371/journal.pone.0155752)
Supplement: S2 Table — (DOCX) [file pone.0155752.s009.docx]

**Table S2. Questions asked in the interview based on Lavides et al. 2010 [13].**

| **Key Areas** | **Key Questions** |
| --- | --- |
| **Demography** | 1. What is your main fishing gear? [type of gear most familiar with and mostly use] |
|  | 1. What and when has the gear changed over the years? |
|  | 1. When did you start fishing? |
|  | 1. How many horsepower is your boat? |
|  | 1. What types of fish species do you target? |
| **Deriving data on Catches** | 1. What types of fish do you usually catch but no longer catch today? |
|  | 1. When did you start to have zero catches of these species in the following decades: 1950s; 1960s; 1970s; 1980s; 1990s; 2000s; current year at time of interview? |
|  | 1. When (1950s; 1960s; 1970s; 1980s; 1990s; 2000s; current year at time of interview) and how great are the previous catches in kilograms per day? |
|  | 1. How many hours do you spend fishing for each fishing trip at the current time of interview and in each of the following decades: 1950s; 1960s; 1970s; 1980s; 1990s; 2000s? |
