# Supplementary material for: Patterns of Coral-Reef Finfish Species Disappearances Inferred from Fishers’ Knowledge in Global Epicentre of Marine Shorefish Diversity
Source: PLoS One. 2016 May 18;11(5):e0155752. doi: 10.1371/journal.pone.0155752 (PMC4871521; doi:10.1371/journal.pone.0155752)
Supplement: S3 Table — (DOCX) [file pone.0155752.s010.docx]

**Table S3. List of species reported to be disappearing from catches.**

| **Common English Name** | **Scientific Name / Local Name** | **Family Name** | **Previous catch rate (kg day^−1^)** |
| --- | --- | --- | --- |
| Bluespine unicornfish | *Naso unicornis* | ACANTHURIDAE | 15 (1980s); 0 (1990s); 0 (2000s); 0 (2012) |
| Yellow and blueback fusilier | *Caesio teres* | CAESIONIDAE | 10 (1960s); 10 (1970s); 5 (1980s); 0 (1990s); 0 (2000s); 0 (2012) |
| African pompano | *Alectis ciliaris* | CARANGIDAE | 1-100 (1950s); 0-100 (1960s);0-100(1970s); 0-700(1980s); 0-200 (1990s); 0-70 (2000s); 0-50 (2014) |
| Golden trevally | *Gnathanodon speciosus* | CARANGIDAE | 1-50 (1950s); 1-50 (1960s); 1-100 (1970s); 0-40 (1980s); 0-250 (1990s); 0-140 (2000s); 0-70 (2014) |
| Subnose pompano | *Trachinotus blochii* | CARANGIDAE | 3 (1950s); 2 (1960s); 1 (1970s); 0 (1980s); 0 (1990s) |
| Oxeye scad | *Selar boops* | CARANGIDAE | 2 (1970s); 2-100 (1980s); 1-200 (1990s); 3-100(2000s); 0-1 (2013) |
| Blue trevally | *Carangoides ferdau* | CARANGIDAE | 2-30 (1960s); 0-20 (1970s); 0-10 (1980s); 0 (1990s); 0 (2000s); 0 (2013) |
| Big eye trevally | *Caranx sexfasciatus* | CARANGIDAE | 3 (1990s); 0-15 (2000s); 0 (2013) |
| Rainbow runner | *Elegatis bipinnulata* | CARANGIDAE | 20 (1970s); 10 (1980s); 2 (1990s); 0 (2000s); 0 (2013) |
| Small spotted dart | *Trachinotus baillonii* | CARANGIDAE | 2 (1950s); 2 (1960s); 1(1970s); 0 (1980s); 0(1990s) |
| Long rakered trevally | *Ulua mentalis* | CARANGIDAE | 1 (1970s); 1 (1980s); 1 (1990s); 0 (2000s); 0 (2012) |
| Brown chub | *Kyphosus bigibbus* | KYPHOSIDAE | 3 (1960s); 30 (1970s); 3-100 (1980s); 0-3 (1990s); 0-2 (2000s); 0 (2012) |
| Blue sea chub | *Kyphosus cinerascens* | KYPHOSIDAE | 3 (1980s); 0 (1990s); 0 (2000s); 0 (2012) |
| Humphead wrasse | *Cheilinus undulatus* | LABRIDAE | 0-50 (1950s); 0-40 (1960s); 0-50 (1970s); 0-100 (1980s); 0-120 (1990s); 0-40 (2000s); 0-40 (2014) |
| Green humphead parrotfish | *Bolbometopon muricatum* | LABRIDAE | 0-90 (1950s); 0-600s (1960s); 0-100 (1970s); 0-300 (1980s); 0-300 (1990s); 0-200 (2000s); 0-50 (2014) |
| Heavybeak parrotfish | *Chlorurus gibbus* | LABRIDAE | 6(1990s); 6(2000s); 0(2014) |
| Steephead parrotfish | *Chlorurus strongylocephalus* | LABRIDAE | 0 (2000s) |
| Ember parrotfish | *Scarus rubroviolaceus* | LABRIDAE | 0 (2000s) |
| Smalltooth emperor | *Lethrinus microdon* | LETHRINIDAE | 1-50 (1950s); 1-600 (1960s); 1-100 (1970s); 0-100 (1980s); 0-200 (1990s); 0-100 (2000s); 0-50 (2014) |
| Mangrove red snapper | *Lutjanus argentimaculatus* | LUTJANIDAE | 1-100 (1950s); 0-60 (1960s); 0-60 (1970s); |
| Chinamanfish | *Symphorus nematophorus* | LUTJANIDAE | 5-18(1960s); 3-9(1970s); 1-12(1980s); 0-10 (1990s); 0-8 (2000s); 0-8(2014) |
| Rusty Jobfish | *Aphareus rutilans* | LUTJANIDAE | 10 (1970s); 6-10(1980s); 4-50 (1990s); 0-50 (2000s); 0-10 (2014) |
| Two-spot red snapper | *Lutjanus bohar* | LUTJANIDAE | 1 (1980s); 1 (1990s); 0-5 (2000s); 0 (2014) |
| Blackspot snapper | *Lutjanus ehrenbergii* | LUTJANIDAE | 2 (1990s); 0 (2000s); 0 (2014) |
| Humphead snapper | *Lutjanus sanguineus* | LUTJANIDAE | 9 (2000s); 0 (2012) |
| Moon fish | *Mene maculata* | MENIDAE | 25 (1950s); 10-25 (1960s); 10-60(1970s); 10-4900 (1980s); 5-4900(1990s); 5-5600 (2000s); 0-4900(2013) |
| Bicolor goatfish | *Parupeneus barberinoides* | MULLIDAE | 8-20 (1950s); 8-20(1960s); 2-20(1970s); 2-40(1980s); 0-65(1990s); 0-8 (2000s); 0-8 (2014) |
| Sulphur goatfish | *Upeneus sulphureus* | MULLIDAE | 5 (1960s); 5 (1970s); 5(1980s); 2 (1990s); 0-70 (2000s); 0 (2012) |
| Dash-and-dot goatfish | *Parupeneus barberinus* | MULLIDAE | 1(2000s); 0 (2012) |
| Giant grouper | *Epinephelus lanceolatus* | SERRANIDAE | 1-300 (1950s); 0-200 (1960s); 0-300 (1970s); 0-105 (1980s); 0-576 (1990s); 0-250 (2000s); 0-62 (2014) |
| Leopard coral grouper | *Plectropomus leopardus* | SERRANIDAE | 5 (1950s); 5 (1960s); 1-50 (1970s); 0-30 (1980s); 0-30 (1990s); 0-20 (2000s); 0-5 (2014) |
| Humpback grouper | *Cromileptes altivelis* | SERRANIDAE | 5 (1950s); 3-5 (1960s); 2-30 (1970s); 1-30 (1980s); 0-8 (1990s); 0-5 (2000s); 0-2 (2014) |
| Orange-spotted grouper | *Epinephelus coioides* | SERRANIDAE | 7 (1950s); 80 (1960s); 0-80 (1970s); 0-80 (1980s); 0-8 (1990s); |
| Brown marbled grouper | *Epinephelus fuscoguttatus* | SERRANIDAE | 0 (1980s) |
| Malabar grouper | *Epinephelus malabaricus* | SERRANIDAE | 2 (2000s); 0 (2012) |
| Black-saddled coralgrouper | *Plectropomus laevis* | SERRANIDAE | 1 (2000s); 0 (2013) |
| Goldlined spinefoot | *Siganus guttatus* | SIGANIDAE | 5-7(1970s); 3-5 (1980s); 2-5 (1990s); 0-2 (2000s); 0-1(2012) |
| Foxface | *Siganus vulpinus* | SIGANIDAE | 1 (1970s); 0 (1980s); 0 (1990s); 0 (2000s) |
| Silver sillago | *Sillago sihama* | SILLAGINIDAE | 2 (1970s); 0 (1980s); 0(1990s); 0(2000s); 0 (2012) |
| Yellowfin seabream | *Acanthopagrus latus* | SPARIDAE | 20(1980s); 10 (1990s); 0 (2000s); 0(2012) |
| Yellowtail barracuda | *Sphyraena flavicauda* | SPHYRAENIDAE | 5(1970s); 0-1(1980s); 0-300 (1990s); 0-8 (2000s); 0-8(2012) |
| Fourlined terapon | *Pelates quadrilineatus* | TERAPONTIDAE | 50 (1970s); 50 (1980s); 30 (1990s); 2-4 (2000s); 0-2 (2012) |
| Unidentified scad species | *Decapterus* sp. | CARANGIDAE | 10-7200 (1970s); 10-7200 (1980s); 4-600 (1990s); 4-600 (2000s); 0-480 (2013) |
| Unidentified jack species | *Caranx sp.* | CARANGIDAE | 2 (1980s); 2(1990s); 0(2000s) |
| Unidentified jack species | Alangulan | CARANGIDAE | 5(1970s;) 5(1980s); 5-20(1990s); 2-20(2000s); 0-1(2013) |
| Unidentified jack species | Haga | CARANGIDAE | 1 (1980s); 1 (2000s); 0 (2012) |
| Unidentified jack species | Lison | CARANGIDAE | 50 (1970s); 20 (1980s); 4-10 (1990s); 0-30 (2000s); 0 (2014) |
| Unidentified sea chub species | Kikilo | KYPHOSIDAE | 10 (1970s); 2 (1980s); 1(1990s); 0 (2000s); 0 (2012) |
| Unidentified pony fish species | Landugan | LEIOGNATHIDAE | 20 (1980s); 10 (1990s); 0 (2000s) |
| Unidentified snapper species | Dampolog | LUTJANIDAE | 15 (1970s); 15 (1980s); 15 (1990s); 0 (2000s); 0 (2012) |
| Unidentified pinecone species | Malapinya | MONOCENTRIDAE | 10 (1950s); 9 (1960s); 7 (1970s); 5 (1980s); 3 (1990s); 2 (2000s); 0 (2012) |
| Local name | Bigik |  | 1 (1950s); 1-30(1960s); 1-30 (1970s); 0-20 (1980s); 0-5 (1990s); 0 (2000s); 0 (2013) |
| Local name | Hurot |  | 12(1960s); 6-15 (1970s); 4-20(1980s); 3-20(1990s); 0-10 (2000s); 0 (2012) |
| Local name | Bakoko |  | 25 (1960s); 25 (1970s); 1 (1980s); 1 (1990s); 0-1 (2000s); 0 (2013) |
| Local name | Surit surit |  | 0(2000s) |
| Local name | Tabaong |  | 1(1990s); 1 (2000s); 0 (2012) |
| Local name | Taewan |  | 1 (1990s); 0(2000s); 0(2012) |
| Local name | Tambangongo |  | 1 (1970s); 0 (1980s); 0 (1990s); 0 (2000s) |
| Local name | Tiktikan |  | 8(1970s); 6 (1980s); 3 (1990s); 0 (2000s) |
