# Supplementary material for: Patterns of Coral-Reef Finfish Species Disappearances Inferred from Fishers’ Knowledge in Global Epicentre of Marine Shorefish Diversity
Source: PLoS One. 2016 May 18;11(5):e0155752. doi: 10.1371/journal.pone.0155752 (PMC4871521; doi:10.1371/journal.pone.0155752)
Supplement: S4 Table — Lmax = maximum body size, Tmat = age at first maturity, k = growth coefficient. (DOCX) [file pone.0155752.s011.docx]

**Table S4. Life History traits of the finfish names identified up to the species level.**

| **Speciesname** | **Family Name** | **Number of fishers reporting 0 catch** | **L_max_ (cm)** | **T_mat_ (yr)** | ***k* (yr^-1^)** | **Trophic level** | **Resilience (yr)** | **Vulnerability** | **IUCNStatus** |
| --- | --- | --- | --- | --- | --- | --- | --- | --- | --- |
| *Bolbometopon muricatum* | LABRIDAE | 301 | 130 | 6.1 | 0.1 | 2.7 ±0.41 se | 4.5 – 14 | 0.67 | VU |
| *Alectis ciliaris* | CARANGIDAE | 263 | 150 | 4.5 | 0.13 | 3.8 ±0.7 se | 4.5 - 14 | 0.67 | LC |
| *Lutjanus argentimaculatus* | LUTJANIDAE | 254 | 150 | 3.2 | 0.19 | 3.6 ±0.6 se | 1.4 - 4.4 | 0.6 | NE |
| *Cheilinus undulatus* | LABRIDAE | 196 | 229 | 5.9 | 0.1 | 4.0 ±0.61 se | 4.5 - 14 | 0.74 | EN |
| *Lethrinus microdon* | LETHRINIDAE | 93 | 80 | 3.2 | 0.2 | 3.8 ±0.53 se | 1.4 - 4.4 | 0.46 | NE |
| *Epinephelus lanceolatus* | SERRANIDAE | 88 | 270 | 13.5 | 0.04 | 4.0 ±0.60 se | >14 | 0.85 | VU |
| *Gnathanodon speciosus* | CARANGIDAE | 64 | 120 | 1.2 | 0.53 | 3.8 ±0.60 se | 1.4 - 4.4 | 0.38 | NE |
| *Eleutheronema tetradactylum* | POLYNEMIDAE | 36 | 200 | 4.3 | 0.13 | 4.4 ±0.8 se | 1.4 - 4.4 | 0.69 | NE |
| *Plectropomus leopardus* | SERRANIDAE | 17 | 120 | 2.5 | 0.25 | 4.5 ±0.8 se | 1.4-4.4 | 0.51 | NT |
| *Cromileptes altivelis* | SERRANIDAE | 13 | 70 | 4 | 0.16 | 4.5   ±0.80 se | 4.5-14 | 0.54 | VU |
| *Epinephelus coioides* | SERRANIDAE | 8 | 120 | 3.7 | 0.17 | 3.9   ±0.7 se | 4.5 - 14 | 0.58 | VU |
| *Trachinotus blochii* | CARANGIDAE | 7 | 110 | 3.6 | 0.17 | 3.7 ±0.46 se | 1.4 - 4.4 | 0.6 | NE |
| *Symphorus nematophorus* | LUTJANIDAE | 6 | 100 | 2.7 | 0.23 | 4.1   ±0.7 se | 1.4 - 4.4 | 0.49 | NE |
| *Kyphosus bigibbus* | KYPHOSIDAE | 5 | 75 | 5.3 | 0.12 | 2.0 ±0.0 se | 4.5 - 14 | 0.6 | NE |
| *Parupeneus barberinoides* | MULLIDAE | 4 | 30 | 1.5 | 0.47 | 3.4   ±0.39 se | <15mo | 0.16 | NE |
| *Selar boops* | CARANGIDAE | 4 | 25 | 0.4 | 1.88 | 3.5 ±0.41 se | <15mo | 0.14 | NE |
| *Caranx melampygous* | CARANGIDAE | 3 | 117 | 2.7 | 0.23 | 4.5 ±0.8 se | 1.4 - 4.4 | 0.5 | NE |
| *Carangoides ferdau* | CARANGIDAE | 3 | 70 | 3 | 0.21 | 4.5   ±0.8 se | 1.4 - 4.4 | 0.44 | NE |
| *Aphareus rutilans* | LUTJANIDAE | 2 | 110 | 3.7 | 0.16 | 4.1   ±0.57 se | 1.4 - 4.4 | 0.58 | NE |
| *Siganus guttatus* | SIGANIDAE | 2 | 42 | 0.4 | 1.66 | 2.7 ±0.30 se | <1.25 | 0.19 | NE |
| *Acanthopagrus latus* | SPARIDAE | 2 | 50 | 3 | 0.23 | 3.2 ±0.5 se | 1.4 - 4.4 | 0.41 | NE |
| *Caranx sexfasciatus* | CARANGIDAE | 2 | 120 | 2.6 | 0.24 | 4.5 ± 0.80  se | 1.4 - 4.4 | 0.45 | LC |
| *Lutjanus bohar* | LUTJANIDAE | 2 | 90 | 7.2 | 0.09 | 4.3   ±0.5 se | 1.4 - 4.4 | 0.69 | NE |
| *Upeneus sulphureus* | MULLIDAE | 2 | 23 | 0.6 | 1.32 | 3.1 ±0.1 se | <15mo | 0.12 | NE |
| *Caranx tille* | CARANGIDAE | 1 | 80 | 2.3 | 0.29 | 4.1 ±0.68 se | 1.4 - 4.4 | 0.38 | NE |
| *Pelates quadrilineatus* | TERAPONTIDAE | 1 | 30 | 2.2 | 0.33 | 3.5 ±0.37 se | <15mo | 0.16 | NE |
| *Ablennes hians* | BELONIDAE | 1 | 140 | 1 | 0.61 | 4.5 ±0.80 se | 1.4 - 4.4 | 0.4 | NE |
| *Selaroides leptolepis* | CARANGIDAE | 1 | 22 | 0.7 | 1.12 | 3.8 ±0.2 se | <15mo | 0.13 | NE |
| *Caesio teres* | CAESIONIDAE | 1 | 40 | 1.5 | 0.46 | 3.4 ±0.45 se | <15 mo | 0.21 | NE |
| *Chlorurus strongylocephalus* | LABRIDAE | 1 | 70 | 2.5 | 0.26 | 2.0 ±0.00 se | 1.4 - 4.4 | 0.39 | NE |
| *Chlorurus gibbus* | LABRIDAE | 1 | 70 | 2.6 | 0.25 | 2.0 ±0.00 se | 4.5 - 14 | 0.39 | LC |
| *Elegatis bipinnulata* | CARANGIDAE | 1 | 180 | 1.4 | 0.21 | 3.6 ±0.49 se | 1.4-4.4 | 0.41 | NE |
| *Epinephelus fuscoguttatus* | SERRANIDAE | 1 | 120 | 3.1 | 0.2 | 4.1   ±0.72 se | 1.4 - 4.4 | 0.5 | NT |
| *Epinephelus malabaricus* | SERRANIDAE | 1 | 234 | 11 | 0.05 | 3.8   ±0.7 se | >14 | 0.4 | NT |
| *Kyphosus cinerascens* | KYPHOSIDAE | 1 | 50 | 2.7 | 0.25 | 2.3 ±0.2 se | 1.4 - 4.4 | 0.38 | NE |
| *Lutjanus ehrenbergii* | LUTJANIDAE | 1 | 35 | 1.7 | 0.42 | 4.4 ±0.8 se | <15mo | 0.19 | NE |
| *Lutjanus sanguineus* | LUTJANIDAE | 1 | 100 | 2.7 | 0.23 | 4.5 ±0.7 se | 4.5 - 14 | 0.47 | NE |
| *Naso unicornis* | ACANTHURIDAE | 1 | 70 | 1.4 | 0.49 | 2.2 ±0.11 se | 4.5 - 14 | 0.32 | LC |
| *Parupeneus barberinus* | MULLIDAE | 1 | 60 | 3.3 | 0.22 | 3.4 ±0.47 se | 1.4 - 4.4 | 0.4 | NE |
| *Plectropomus laevis* | SERRANIDAE | 1 | 125 | 14 | 0.1 | 4.1 ±0.57 se | >14 | 0.72 | VU |
| *Scarus rubroviolaceus* | LABRIDAE | 1 | 70 | 3.9 | 0.17 | 2.0 ±0.00 se | 1.4 - 4.4 | 0.52 | LC |
| *Siganus vulpinus* | SIGANIDAE | 1 | 25 | 1.1 | 0.67 | 2.7 ±0.30 se | <1.25 | 0.23 | NE |
| *Sillago sihama* | SILLAGINIDAE | 1 | 31 | 1 | 0.75 | 3.3 ±0.1 se | <15mo | 0.22 | NE |
| *Terapon jarbua* | TERAPONTIDAE | 1 | 36 | 3.1 | 0.23 | 3.9 ±0.5 se | 1.4 - 4.4 | 0.37 | LC |
| *Trachinotus baillonii* | CARANGIDAE | 1 | 60 | 1.9 | 0.34 | 3.6   ±0.57 se | 1.4-4.4 | 0.35 | NE |
| *Ulua mentalis* | CARANGIDAE | 1 | 100 | 2.9 | 0.21 | 3.7 ±0.51 se | 1.4 - 4.4 | 0.42 | NE |
| *Pterocaesio digramma* | CAESIONIDAE | 1 | 30 | 2.5 | 0.3 | 3.4 ±0.48 se | 1.4 - 4.4 | 0.31 | NE |
| *Decapterus macarellus* | CARANGIDAE | 1 | 46 | 0.9 | 0.8 | 4.0 ±0.2 se | <15mo | 0.23 | NE |

L_max_= maximum body size, T_mat_=age at first maturity, *k*= growth coefficient.
