# Supplementary material for: Patterns of Coral-Reef Finfish Species Disappearances Inferred from Fishers’ Knowledge in Global Epicentre of Marine Shorefish Diversity
Source: PLoS One. 2016 May 18;11(5):e0155752. doi: 10.1371/journal.pone.0155752 (PMC4871521; doi:10.1371/journal.pone.0155752)
Supplement: S5 Table — (DOCX) [file pone.0155752.s012.docx]

**Table S5.** **Main fishing gears used by fishers per marine KBA.**

| **Main Fishing Gear** | **Danajon Bank** | **Lanuza Bay** | **Honda Bay** | **Polillo** | **VIP** | **Total** |
| --- | --- | --- | --- | --- | --- | --- |
| Gillnet | 454 | 139 | 159 | 64 | 239 | 1055 |
| Hook and line | 216 | 171 | 195 | 244 | 165 | 991 |
| Spear | 127 | 84 | 58 | 55 | 12 | 336 |
| Compressor fishing | 59 |  | 1 | 14 |  | 74 |
| Pots | 50 | 4 |  |  |  | 54 |
| Fish corral | 28 | 9 | 5 | 3 | 2 | 47 |
| Aquarium gillnet |  |  |  | 2 | 37 | 39 |
| Danish seine | 18 |  |  |  |  | 18 |
| Dynamite |  |  |  | 11 |  | 11 |
| Fish cage |  |  | 1 | 8 |  | 9 |
| Cyanide |  |  |  | 4 |  | 4 |
| Beach seine |  | 4 |  |  |  | 4 |
| Stationary lift net |  |  | 3 |  |  | 3 |
| Trawl | 3 |  |  |  |  | 3 |
