# Supplementary material for: Patterns of Coral-Reef Finfish Species Disappearances Inferred from Fishers’ Knowledge in Global Epicentre of Marine Shorefish Diversity
Source: PLoS One. 2016 May 18;11(5):e0155752. doi: 10.1371/journal.pone.0155752 (PMC4871521; doi:10.1371/journal.pone.0155752)
Supplement: S6 Table — (DOCX) [file pone.0155752.s013.docx]

**Table S6.** **Target species of fishers in each marine KBA.**

| **Target species** | **Danajon Bank** | **Lanuza Bay** | **Honda Bay** | **Polillo** | **VIP** | **Total** |
| --- | --- | --- | --- | --- | --- | --- |
| Reef-associated fishes | 794 | 258 | 335 | 379 | 237 | 2003 |
| Pelagic | 132 | 102 | 50 | 21 | 127 | 432 |
| Tuna and tuna-like fishes | 3 | 6 | 1 | 4 | 24 | 38 |
| All of the above | 26 | 45 | 36 | 1 | 67 | 175 |
