# Supplementary material for: Patterns of Coral-Reef Finfish Species Disappearances Inferred from Fishers’ Knowledge in Global Epicentre of Marine Shorefish Diversity
Source: PLoS One. 2016 May 18;11(5):e0155752. doi: 10.1371/journal.pone.0155752 (PMC4871521; doi:10.1371/journal.pone.0155752)
Supplement: S7 Table — (DOCX) [file pone.0155752.s014.docx]

**Table S7. Variables included in initial models used in GLMM Analysis.**

| Variable | Description |
| --- | --- |
| *Temporal* |  |
| Decade | 1950s, 1960s, 1970s, 1980s, 1990s, 2000s, 2012 (Lanuza Bay and Danajon Bank), 2013 (Verde Island Passage and Polillo), 2014 (Honda Bay) |
| *Demography* |  |
| IntervieweeID | Random number tagged per interviewee |
| Age | 21-97 years old |
| AgeDecade^2^ | Square of decadal age |
| mKBA | Lanuza Bay, Danajon Bank, Verde Island Passage, Polillo and Honda Bay (see Table S1) |
| *Fishing Practices* |  |
| Gear | Gillnet, Hook and Line, Spear, Compressor Fishing, Pots, Fish Corral, Aquarium Gillnet, Danish Seine, Dynamite, Fish Cage, Cyanide, Beach Seine, Stationary Lift net, Trawl (see Table S5) |
| Hours Fishing | 1-52 hours |
| Horsepower | 0-80hp |
| Fishing Experience | 1-81 years |
